# Supplementary material for: Phenotypic clines in herbivore resistance and reproductive traits in wild plants along an agricultural gradient
Source: PLoS One. 2023 May 31;18(5):e0286050. doi: 10.1371/journal.pone.0286050 (PMC10231797; doi:10.1371/journal.pone.0286050)
Supplement: S1 File — (DOCX) [file pone.0286050.s001.docx]

**Statistics for Table S3, S4, and S5 and Figures S1, S2, and S3**

To determine the most predictive land use classification and spatial scale across all evaluated traits per species we used the dredge function (R package MuMln; Bartoń, 2013) to construct all possible mixed effects models at each scale (500, 1000, and 1500m) using the nlme package (Pinheiro et al., 2014). Raw trait values were scaled so that all traits were weighted evenly in the model. We created models for each species individually, including all traits in a single model. Here we included the interaction between traits and landscape type, with parent plant nested within collection site as random effects. The land use classification and scale with the lowest AICc score was identified separately for each species and used in all further analyses.

To evaluate changes in trait expression across the land use gradient we fit separate linear mixed models using the R package lme4 with each trait as the response variable and the landscape variable described in the previous analysis as a fixed effect. We ran individual models for each species and within each species separate models were fit for each trait (field collected seed mass, germination rate, petal area, anther-stigma distance, plant mass, self-pollination rate, self-pollination seed mass, caterpillar consumption efficiency, leaf area consumed, and caterpillar relative growth rate). Each model included a nested random effect of parent plant to control for multiple offspring from each parent nested within collection site to control for psuedoreplication of multiple parents within a site. Models for field collected seed mass and germination rate only included collection site as a random effect. For models evaluating petal size, we included plant mass as a predictor to control for variation in plant size. In each model evaluating *T. arvense* reproductive traits (petal area, seed mass, plant mass) we included vernalization as a random effect. We used a logistic regression to test if caterpillar survival correlated significantly with the landscape gradient. Given the low mortality overall, we excluded any caterpillars that didn’t survive to the end of the experiment in all future models. In the models evaluating defensive traits from the bioassay (caterpillar consumption efficiency, leaf area consumed, and caterpillar relative growth rate), we included bolting status as a random effect for *T. arvense* and *C. bursa-pastoris.* For all models, we evaluated the significance of individual trait responses using a type three anova.
