# Supplementary material for: Phenotypic clines in herbivore resistance and reproductive traits in wild plants along an agricultural gradient
Source: PLoS One. 2023 May 31;18(5):e0286050. doi: 10.1371/journal.pone.0286050 (PMC10231797; doi:10.1371/journal.pone.0286050)
Supplement: S2 File — (DOCX) [file pone.0286050.s002.docx]

**Cornell Mix Recipe**

Add:

2 bales 3.8 Peat Moss tumbled

Add:

4 lbs. Calcium Sulfate

4 lbs. Jacks professional media mix 111 10-5-10

5 lbs. Lime

.5 lbs. Wetting Agent = 1 4” pot

Tumble until well mixed.

Add:

Water

Add:

1 Perlite bag

2 Vermiculite bags.

Mix Well. Water more if needed.
