# Supplementary material for: Phenotypic clines in herbivore resistance and reproductive traits in wild plants along an agricultural gradient
Source: PLoS One. 2023 May 31;18(5):e0286050. doi: 10.1371/journal.pone.0286050 (PMC10231797; doi:10.1371/journal.pone.0286050)
Supplement: S1 Fig — Germination rate (A) and seed mass (B) of seeds collected from B. vulgaris plants growing at sites representing a gradient of increasing open (non-forested) natural land cover in a 1000m radius around the collection site. Petal area (C), plant mass (D), self-pollinated seed mass (E), percent aborted seed pods (F), and stigma-anther distance (G) of the offspring grown from the parent populations in a greenhouse common garden. Caterpillar consumed leaf area (H) and consumption efficiency (I) from a no-choice herbivore bioassay represent a proxy for plant defense traits in the offspring grown from the parent populations. Solid regression lines indicate significant correlations, dashed indicate marginal correlations, and no regression line indicates no relationship. Error bars represent standard error. (DOCX) [file pone.0286050.s003.docx]

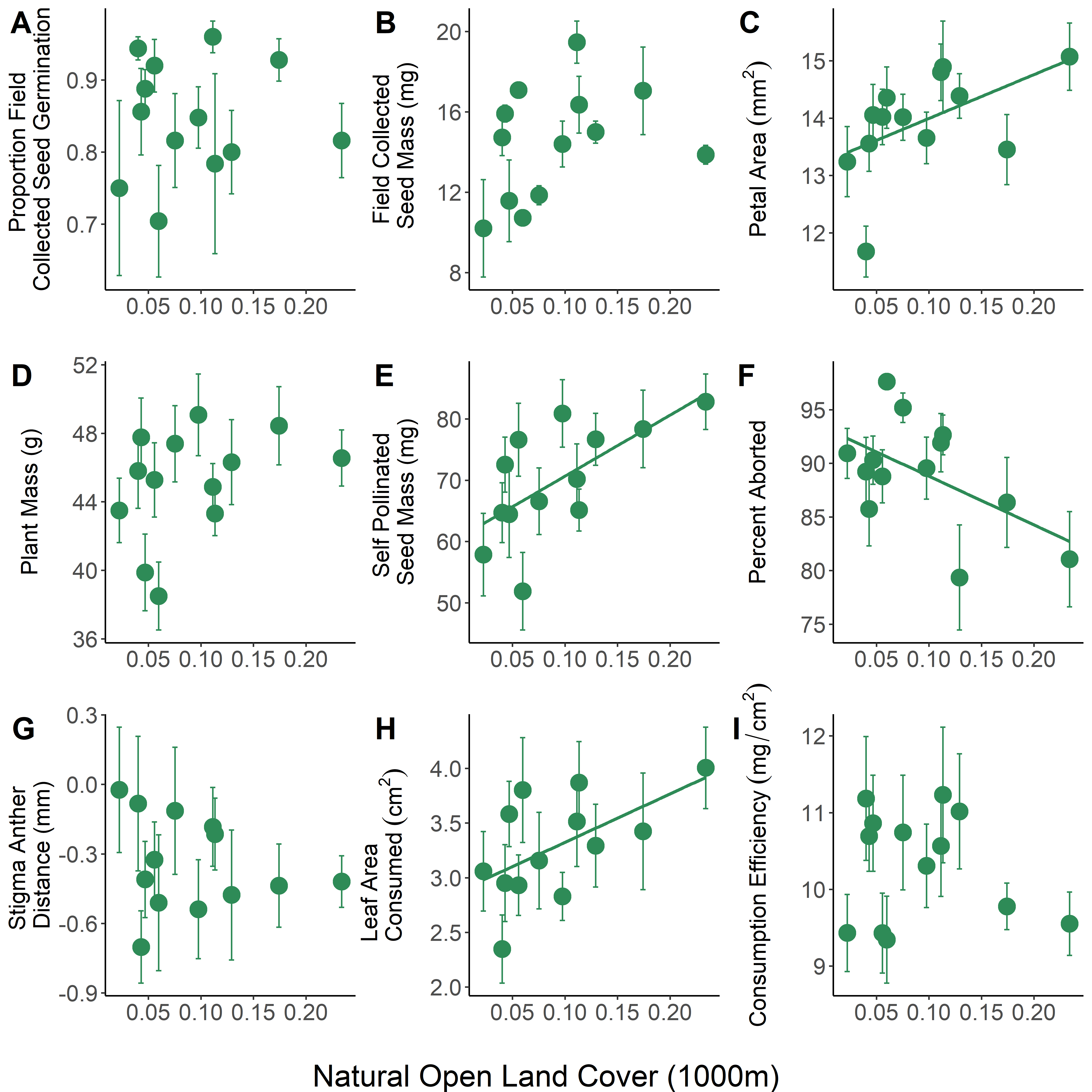


**S1 Fig.** Germination rate (A) and seed mass (B) of seeds collected from *B. vulgaris* plants growing at sites representing a gradient of increasing open (non-forested) natural land cover in a 1000m radius around the collection site. Petal area (C), plant mass (D), self-pollinated seed mass (E), percent aborted seed pods (F), and stigma-anther distance (G) of the offspring grown from the parent populations in a greenhouse common garden. Caterpillar consumed leaf area (H) and consumption efficiency (I) from a no-choice herbivore bioassay represent a proxy for plant defense traits in the offspring grown from the parent populations. Solid regression lines indicate significant correlations, dashed indicate marginal correlations, and no regression line indicates no relationship. Error bars represent standard error.
