# Supplementary material for: Phenotypic clines in herbivore resistance and reproductive traits in wild plants along an agricultural gradient
Source: PLoS One. 2023 May 31;18(5):e0286050. doi: 10.1371/journal.pone.0286050 (PMC10231797; doi:10.1371/journal.pone.0286050)
Supplement: S1 Table — (DOCX) [file pone.0286050.s006.docx]

**S1 Table.** CDL land cover types included within the broad land use classifications used in the PCA in the main text and individual analyses included in the supplement.

| **Land Cover Classification** | **CDL Land Cover Type** |
| --- | --- |
| Agriculture | - Corn - Sorghum - Soybeans - Sweet corn - Barely - Spring wheat - Winter wheat - Rye - Oats - Alfalfa - Buckwheat - Dry beans - Other Crops - Miscellaneous vegetables and fruits - Onion - Peas - Pears - Fallow/Idle Cropland - Triticale - Squash - Pumpkins - Cabbage - Double Crop Oats/corn - Peaches - Apples - Grapes - Christmas Trees - Clover Wildflowers |
| Pasture | - Other Hay Non-alfalfa - Grassland Pasture |
| Natural Open | - Clover Wildflowers - Shrubland - Herbaceous Wetlands - Developed Open Space |
| Natural Forested | - Deciduous Forest - Evergreen Forest - Mixed Forest - Woody Wetlands |
| Developed | - Developed Low Intensity - Developed Medium Intensity - Developed High Intensity |
| Natural (Open and Forested) | - Clover Wildflowers - Shrubland - Herbaceous Wetlands - Developed Open Space - Deciduous Forest - Evergreen Forest - Mixed Forest - Woody Wetlands |
| Open Natural and Pasture | - Clover Wildflowers - Shrubland - Herbaceous Wetlands - Developed Open Space - Other Hay Non-alfalfa - Grassland Pasture |
| Agriculture and Pasture | - Corn - Sorghum - Soybeans - Sweet corn - Barely - Spring wheat - Winter wheat - Rye - Oats - Alfalfa - Buckwheat - Dry beans - Other Crops - Miscellaneous vegetables and fruits - Onion - Peas - Pears - Fallow/Idle Cropland - Triticale - Squash - Pumpkins - Cabbage - Double Crop Oats/corn - Peaches - Apples - Grapes - Christmas Trees - Clover Wildflowers - Other Hay Non-alfalfa - Grassland Pasture |
