# Supplementary material for: Phenotypic clines in herbivore resistance and reproductive traits in wild plants along an agricultural gradient
Source: PLoS One. 2023 May 31;18(5):e0286050. doi: 10.1371/journal.pone.0286050 (PMC10231797; doi:10.1371/journal.pone.0286050)
Supplement: S2 Table — Statistically significant predictors (P < 0.05) are indicated in bold. (DOCX) [file pone.0286050.s007.docx]

**S2 Table.** Results of Mantel test^1^ of spatial autocorrelation in the residuals of the final models. Statistically significant predictors (*P* < 0.05) are indicated in bold.

| **Species** | **Trait** | **Monte-Carlo test Observation** | **p-value** |
| --- | --- | --- | --- |
| B. vulgaris | Field Collected Seed Mass | -0.03024187 | 0.9218 |
|  | Germination Rate | -0.01838491 | 0.7617 |
|  | Petal Area | -0.039 | 0.9286 |
|  | Stigma-anther distance | 0.002376388 | 0.2966 |
|  | **Self-Pollination Rate** | **0.01207192** | **0.0159** |
|  | Self-Pollinated Seed Mass | -0.001726054 | 0.6284 |
|  | Plant Mass | -0.003470359 | 0.7375 |
|  | Leaf Area Consumed | 0.001751181 | 0.3613 |
|  | Relative Growth Rate | 0.007724084 | 0.1087 |
|  | Consumption Efficiency | 0.004272711 | 0.2456 |
|  |  |  |  |
| T. arvense | Field Collected Seed Mass | 0.008273677 | 0.3716 |
|  | Germination Rate | 0.02411333 | 0.1216 |
|  | Petal Area | 0.0107605 | 0.1974 |
|  | Stigma-anther distance | 0.00271706 | 0.35 |
|  | Self-Pollination Rate | -0.0004034195 | 0.5143 |
|  | Self-Pollinated Seed Mass | -0.002564153 | 0.6597 |
|  | Plant Mass | -0.006316432 | 0.8596 |
|  | Leaf Area Consumed | -0.0122374 | 0.98 |
|  | Relative Growth Rate | -0.006106258 | 0.8165 |
|  | Consumption Efficiency | -0.007598068 | 0.8449 |
|  |  |  |  |
| C. bursa-pastoris | Field Collected Seed Mass | -0.05510 | 0.9275 |
|  | Germination Rate | -0.09668 | 1 |
|  | Petal Area | -0.04755 | 0.9316 |
|  | Stigma-anther distance | -0.003739 | 0.6879 |
|  | Self-Pollination Rate | 0.007866 | 0.1579 |
|  | Self-Pollinated Seed Mass | -0.005944642 | 0.7871 |
|  | Plant Mass | -0.00804 | 0.8769 |
|  | Leaf Area Consumed | -0.00926 | 0.9784 |
|  | Relative Growth Rate | -0.00367 | 0.7552 |
|  | Consumption Efficiency | 0.002847 | 0.2873 |

1. All final models were tested for spatial autocorrelation in the residuals using the mantel test from the package ade4 (Dray & Dufour, 2007).
