# Supplementary material for: Phenotypic clines in herbivore resistance and reproductive traits in wild plants along an agricultural gradient
Source: PLoS One. 2023 May 31;18(5):e0286050. doi: 10.1371/journal.pone.0286050 (PMC10231797; doi:10.1371/journal.pone.0286050)
Supplement: S3 Table — Parent plant nested within collection site was included as a random effect in all models. Statistically significant predictors (P < 0.05) are indicated in bold and marginal predictors (P < 0.1) are italicized. Trichoplusia ni caterpillars used in the leaf bioassay are included in the predictor column as T.ni. (DOCX) [file pone.0286050.s008.docx]

| Trait | N Plants | Predictor | Chisq | Df | p-value |
| --- | --- | --- | --- | --- | --- |
| Field Collected Germination | 63 | Natural Land Cover | 0.0268 | 1 | 0.87 |
| Field Collected Seed Mass | 63 | Natural Land Cover | 1.4956 | 1 | 0.2214 |
| **Petal Area** | **305** | **Natural Land Cover** | **3.8915** | **1** | **0.0485** |
|  |  | Plant Mass | 0.0073 | 1 | 0.9317 |
| Plant Mass | 307 | Natural Land Cover | 1.7833 | 1 | 0.1817 |
| **Self-Pollinated Seed Mass** | **306** | **Natural Land Cover** | **9.083** | **1** | **0.0026** |
|  |  | **Plant Mass** | **67.251** | **1** | **<0.0001** |
| **Proportion Aborted Seed Pods** | **301** | **Natural Land Cover** | **4.2643** | **1** | **0.0389** |
| Stigma-Anther Distance | 308 | Natural Land Cover | 0.2783 | 1 | 0.5978 |
| **Consumed Leaf Area** | **234** | **Natural Land Cover** | **3.5577** | **1** | **0.0593** |
|  |  | ***T. ni* Initial Mass** | **55.796** | **1** | **<0.0001** |
|  |  | **Leaf Size** | **6.1773** | **1** | **0.0129** |
| Caterpillar Consumption Efficiency | 243 | Natural Land Cover | 0.4200 | 1 | 0.5169 |
|  |  | ***T. ni* Initial Mass** | **5.3758** | **1** | **0.0204** |
|  |  | *Leaf Size* | *2.9705* | *1* | *0.0848* |
| Relative Growth Rate | 269 | Natural Land Cover | 0.7189 | 1 | 0.3965 |
|  |  | *Leaf Size* | *3.3813* | *1* | *0.0659* |

**S3 Table**. Results of general linear mixed models investigating the effects of open natural land cover on all measured traits for *B. vulgaris*. Parent plant nested within collection site was included as a random effect in all models. Statistically significant predictors (*P* < 0.05) are indicated in bold and marginal predictors (*P* < 0.1) are italicized. *Trichoplusia ni* caterpillars used in the leaf bioassay are included in the predictor column as *T.ni*.
