# Supplementary material for: Phenotypic clines in herbivore resistance and reproductive traits in wild plants along an agricultural gradient
Source: PLoS One. 2023 May 31;18(5):e0286050. doi: 10.1371/journal.pone.0286050 (PMC10231797; doi:10.1371/journal.pone.0286050)
Supplement: S4 Table — Parent plant nested within collection site was included as a random effect in all models. Statistically significant predictors (P < 0.05) are indicated in bold and marginal predictors (P < 0.1) are italicized. Trichoplusia ni caterpillars used in the leaf bioassay are included in the predictor column as T.ni. (DOCX) [file pone.0286050.s009.docx]

**S4 Table**. Results of general linear mixed models investigating the effects of agricultural land cover on all measured traits for *C. bursa-pastoris*. Parent plant nested within collection site was included as a random effect in all models. Statistically significant predictors (*P* < 0.05) are indicated in bold and marginal predictors (*P* < 0.1) are italicized. *Trichoplusia ni* caterpillars used in the leaf bioassay are included in the predictor column as *T.ni*.

| Trait | N Plants | N Sites | Predictor | Chisq | Df | p-value |
| --- | --- | --- | --- | --- | --- | --- |
| **Field Collected Germination** | **78** | **16** | **Ag Land Cover** | **11.778** | **1** | **0.0006** |
| Field Collected Seed Mass | 78 | 16 | Ag Land Cover | 0.1737 | 1 | 0.6769 |
| **Petal Area** | **196** | **16** | **Ag Land Cover** | **5.1977** | **1** | **0.0226** |
|  |  |  | *Plant Mass* | *2.7833* | *1* | *0.0953* |
| Plant Mass | 209 | 16 | Ag Land Cover | 1.234 | 1 | 0.2666 |
| **Self-Pollinated Seed Mass** | **200** | **16** | **Ag Land Cover** | **6.0518** | **1** | **0.0139** |
|  |  |  | Plant Mass | 1.9130 | 1 | 0.1666 |
| Proportion Aborted Seed Pods | 219 | 16 | Ag Land Cover | 0.9808 | 1 | 0.322 |
| Stigma-Anther Distance | 216 | 16 | Ag Land Cover | 1.2860 | 1 | 0.2568 |
| **Consumed Leaf Area** | **288** | **16** | **Ag Land Cover** | **6.2172** | **1** | **0.0127** |
|  |  |  | ***T. ni* Initial Mass** | **29.102** | **1** | **<0.0001** |
|  |  |  | **Leaf Size** | **27.431** | **1** | **<0.0001** |
| **Caterpillar Consumption Efficiency** | **287** | **16** | **Ag Land Cover** | **4.7200** | **1** | **0.0298** |
|  |  |  | *T. ni* Initial Mass | 2.6206 | 1 | 0.1055 |
|  |  |  | **Leaf Size** | **18.834** | **1** | **<0.0001** |
| Relative Growth Rate | 287 | 16 | Ag Land Cover | 0.6722 | 1 | 0.4123 |
|  |  |  | **Leaf Size** | **6.2724** | **1** | **0.0123** |
