# Supplementary material for: Phenotypic clines in herbivore resistance and reproductive traits in wild plants along an agricultural gradient
Source: PLoS One. 2023 May 31;18(5):e0286050. doi: 10.1371/journal.pone.0286050 (PMC10231797; doi:10.1371/journal.pone.0286050)
Supplement: S5 Table — Parent plant nested within collection site was included as a random effect in all models. Statistically significant predictors (P < 0.05) are indicated in bold and marginal predictors (P < 0.1) are italicized. Trichoplusia ni caterpillars used in the leaf bioassay are included in the predictor column as T.ni. (DOCX) [file pone.0286050.s010.docx]

**S5 Table**. Results of general linear mixed models investigating the effects of pasture land cover on all measured traits for *C. bursa-pastoris*. Parent plant nested within collection site was included as a random effect in all models. Statistically significant predictors (*P* < 0.05) are indicated in bold and marginal predictors (*P* < 0.1) are italicized. *Trichoplusia ni* caterpillars used in the leaf bioassay are included in the predictor column as *T.ni*.

| Trait | N Plants | Predictor | Chisq | Df | p-value |
| --- | --- | --- | --- | --- | --- |
| Field Collected Germination | 71 | Pasture Land Cover | 0.2309 | 1 | 0.6308 |
| **Field Collected Seed Mass** | **71** | **Pasture Land Cover** | **5.2025** | **1** | **0.0226** |
| Petal Area | 250 | Pasture Land Cover | 0.9428 | 1 | 0.3315 |
|  |  | Plant Mass | 0.3090 | 1 | 0.5783 |
| Plant Mass | 269 | Pasture Land Cover | 1.0121 | 1 | 0.3144 |
| **Self-Pollinated Seed Mass** | **237** | **Pasture Land Cover** | **3.8914** | **1** | **0.0485** |
|  |  | **Plant Mass** | **81.959** | **1** | **<0.0001** |
| Proportion Aborted Seed Pods | 271 | Pasture Land Cover | 0.0018 | 1 | 0.9666 |
| Stigma-Anther Distance | 256 | Pasture Land Cover | 0.0002 | 1 | 0.9885 |
| Consumed Leaf Area | 219 | *Pasture Land Cover* | *2.9296* | *1* | *0.0870* |
|  |  | ***T. ni* Initial Mass** | **4.3993** | **1** | **0.0360** |
|  |  | **Leaf Size** | **78.711** | **1** | **<0.0001** |
| **Caterpillar Consumption Efficiency** | **217** | **Pasture Land Cover** | **5.2919** | **1** | **0.0214** |
|  |  | ***T. ni* Initial Mass** | **4.4647** | **1** | **0.0346** |
|  |  | Leaf Size | 0.5154 | 1 | 0.4728 |
| **Relative Growth Rate** | **216** | **Pasture Land Cover** | **8.2299** | **1** | **0.0041** |
|  |  | **Leaf Size** | **24.468** | **1** | **<0.0001** |
