# Supplementary material for: Phenotypic clines in herbivore resistance and reproductive traits in wild plants along an agricultural gradient
Source: PLoS One. 2023 May 31;18(5):e0286050. doi: 10.1371/journal.pone.0286050 (PMC10231797; doi:10.1371/journal.pone.0286050)
Supplement: Attachment — Submitted filename: PONE-D-23-01036 in rev.pdf [file pone.0286050.s011.pdf]

PLOS ONE

Phenotypic clines in herbivore resistance and reproductive traits in wild plants along an agricultural gradient

--Manuscript Draft--

**Manuscript Number:**	PONE-D-23-01036
**Article Type:**	Research Article
**Full Title:**	Phenotypic clines in herbivore resistance and reproductive traits in wild plants along an agricultural gradient
**Short Title:**	Phenotypic clines in wild plant traits along an agricultural gradient
**Corresponding Author:**	Hayley Schroeder<br>Cornell University College of Agriculture and Life Sciences<br>Ithaca, NY UNITED STATES
**Keywords:**	Landscape Simplification, Plant-Insect Interactions, Plant Traits, Anthropogenic Change, Local Adaptation, Land-use Change
**Abstract:**	The conversion of natural landscapes to agriculture is a leading cause of biodiversity loss worldwide. While many studies examine how landscape modification affects species diversity, a trait-based approach can provide new insights into species responses to environmental change. Wild plants persisting in heavily modified landscapes provide a unique opportunity to examine species' responses to land use change. Trait expression within a community plays an important role in structuring species interactions, highlighting the potential implications of landscape mediated trait changes on ecosystem functioning. Here we test the effect of increasing agricultural landscape modification on defensive and reproductive traits in three commonly occurring Brassicaceae species to evaluate plant responses to landscape change. We collected seeds from populations at spatially separated sites with variation in surrounding agricultural land cover and grew them in a greenhouse common garden, measuring defensive traits through an herbivore no-choice bioassay as well as reproductive traits such as flower size and seed set. In two of the three species, plants originating from agriculturally dominant landscapes expressed a consistent reduction in flower size and herbivore leaf consumption. One species also showed reduced fitness associated with increasingly agricultural landscapes. These findings suggest that wild plants are responding to landscape modification, highlighting that species diversity alone does not fully capture the effects of land use change.
**Order of Authors:**	Hayley Schroeder
	Heather Grab
	Katja Poveda
**Additional Information:**	
**Question**	**Response**
**Financial Disclosure**<br><br>Enter a financial disclosure statement that describes the sources of funding for the work included in this submission. Review the submission guidelines for detailed requirements. View published research articles from *PLOS ONE* for specific examples.<br><br>This statement is required for submission and **will appear in the published article** if the submission is accepted. Please make	HS was supported by the Cornell University's Presidential Life Sciences Fellowship. Research was funded by the Cornell Entomology Griswold Endowment and the Atkinson Center Sustainable Biodiversity Fund. The funders had no role in study design, data collection and analysis, decision to publish, or preparation of the manuscript.

Powered by Editorial Manager® and ProduXion Manager® from Aries Systems Corporation

sure it is accurate.<br><br>**Unfunded studies**<br>Enter: *The author(s) received no specific funding for this work.*<br><br>**Funded studies**<br>Enter a statement with the following details:<br>• Initials of the authors who received each award<br>• Grant numbers awarded to each author<br>• The full name of each funder<br>• URL of each funder website<br>• Did the sponsors or funders play any role in the study design, data collection and analysis, decision to publish, or preparation of the manuscript?<br>• **NO** - Include this sentence at the end of your statement: *The funders had no role in study design, data collection and analysis, decision to publish, or preparation of the manuscript.*<br>• **YES** - Specify the role(s) played.<br><br>* typeset	
**Competing Interests**<br><br>Use the instructions below to enter a competing interest statement for this submission. On behalf of all authors, disclose any competing interests that could be perceived to bias this work—acknowledging all financial support and any other relevant financial or non-financial competing interests.<br><br>This statement is required for submission and **will appear in the published article** if the submission is accepted. Please make sure it is accurate and that any funding sources listed in your Funding Information later in the submission form are also declared in your Financial Disclosure statement.<br><br>View published research articles from *PLOS ONE* for specific examples.	The authors have declared that no competing interests exist.

**NO authors have competing interests**<br><br>Enter: *The authors have declared that no competing interests exist.*<br><br>**Authors with competing interests**<br><br>Enter competing interest details beginning with this statement:<br><br>*I have read the journal's policy and the authors of this manuscript have the following competing interests: [insert competing interests here]*<br><br>* typeset	
**Ethics Statement**<br><br>Enter an ethics statement for this submission. This statement is required if the study involved:<br><br>• Human participants<br>• Human specimens or tissue<br>• Vertebrate animals or cephalopods<br>• Vertebrate embryos or tissues<br>• Field research<br><br>Write "N/A" if the submission does not require an ethics statement.<br><br>General guidance is provided below. Consult the submission guidelines for detailed instructions. **Make sure that all information entered here is included in the Methods section of the manuscript.**	N/A

Powered by Editorial Manager® and ProduXion Manager® from Aries Systems Corporation

**Format for specific study types**<br><br>**Human Subject Research (involving human participants and/or tissue)**<br>• Give the name of the institutional review board or ethics committee that approved the study<br>• Include the approval number and/or a statement indicating approval of this research<br>• Indicate the form of consent obtained (written/oral) or the reason that consent was not obtained (e.g. the data were analyzed anonymously)<br><br>**Animal Research (involving vertebrate animals, embryos or tissues)**<br>• Provide the name of the Institutional Animal Care and Use Committee (IACUC) or other relevant ethics board that reviewed the study protocol, and indicate whether they approved this research or granted a formal waiver of ethical approval<br>• Include an approval number if one was obtained<br>• If the study involved *non-human primates*, add *additional details* about animal welfare and steps taken to ameliorate suffering<br>• If anesthesia, euthanasia, or any kind of animal sacrifice is part of the study, include briefly which substances and/or methods were applied<br><br>**Field Research**<br><br>Include the following details if this study involves the collection of plant, animal, or other materials from a natural setting:<br>• Field permit number<br>• Name of the institution or relevant body that granted permission	
**Data Availability**<br><br>Authors are required to make all data underlying the findings described fully available, without restriction, and from the time of publication. PLOS allows rare exceptions to address legal and ethical concerns. See the PLOS Data Policy and FAQ for detailed information.	Yes - all data are fully available without restriction

A Data Availability Statement describing where the data can be found is required at submission. Your answers to this question constitute the Data Availability Statement and **will be published in the article**, if accepted.<br><br>**Important:** Stating 'data available on request from the author' is not sufficient. If your data are only available upon request, select 'No' for the first question and explain your exceptional situation in the text box.<br><br>Do the authors confirm that all data underlying the findings described in their manuscript are fully available without restriction?	
**Describe where the data may be found in full sentences. If you are copying our sample text, replace any instances of XXX with the appropriate details.**<br><br>• If the data are **held or will be held in a public repository**, include URLs, accession numbers or DOIs. If this information will only be available after acceptance, indicate this by ticking the box below. For example: *All XXX files are available from the XXX database (accession number(s) XXX, XXX.).*<br>• If the data are all contained **within the manuscript and/or Supporting Information files**, enter the following: *All relevant data are within the manuscript and its Supporting Information files.*<br>• If neither of these applies but you are able to provide **details of access elsewhere**, with or without limitations, please do so. For example:<br><br>*Data cannot be shared publicly because of [XXX]. Data are available from the XXX Institutional Data Access / Ethics Committee (contact via XXX) for researchers who meet the criteria for access to confidential data.*<br><br>*The data underlying the results presented in the study are available from (include the name of the third party*	All 11 files are available from the Dryad Repository (DOI https://doi.org/10.5061/dryad.905qfttq6).

and contact information or URL).<br>• This text is appropriate if the data are owned by a third party and authors do not have permission to share the data.<br><br>* typeset	
Additional data availability information:	

Powered by Editorial Manager® and ProduXion Manager® from Aries Systems Corporation

Manuscript

Click here to access/download;Manuscript;PLOS_ONE_Main_Text_No_Figure

Full Title: Phenotypic clines in herbivore resistance and reproductive traits in wild plants along an agricultural gradient

Short Title: Phenotypic clines in wild plant traits along an agricultural gradient

Hayley Schroeder[1*], Heather Grab[2], Katja Poveda[1]

[1]Department of Entomology, Cornell University, Ithaca, NY, United States
[2]School of Integrative Plant Sciences, Cornell University, Ithaca, NY, United States

*Corresponding author

E-mail: hayleyadair37@gmail.com

Abstract

The conversion of natural landscapes to agriculture is a leading cause of biodiversity loss worldwide. While many studies examine how landscape modification affects species diversity, a trait-based approach can provide new insights into species responses to environmental change. Wild plants persisting in heavily modified landscapes provide a unique opportunity to examine species' responses to land use change. Trait expression within a community plays an important role in structuring species interactions, highlighting the potential implications of landscape mediated trait changes on ecosystem functioning. Here we test the effect of increasing agricultural landscape modification on defensive and reproductive traits in three commonly occurring Brassicaceae species to evaluate plant responses to landscape change. We collected seeds from populations at spatially separated sites with variation in surrounding agricultural land cover and grew them in a greenhouse common garden, measuring defensive traits through an herbivore no-choice bioassay as well as reproductive traits such as flower size and seed set. In two of the three species, plants originating from agriculturally dominant landscapes expressed a consistent reduction in flower size and herbivore leaf consumption. One species also showed reduced fitness associated with increasingly agricultural landscapes. These findings suggest that wild plants are responding to landscape modification, highlighting that species diversity alone does not fully capture the effects of land use change.

Keywords: Landscape Simplification, Plant-Insect Interactions, Plant Traits, Anthropogenic Change, Local Adaptation, Land-use Change

Introduction

Though habitat change is a natural part of global ecosystems, the scale and intensity of anthropogenic habitat disturbance in the last hundred years is unprecedented (1,2). In much of the world, human modified landscapes are frequently characterized by expansive monocultures with intensive chemical and mechanical management. Within these heavily modified landscapes, semi-natural habitat patches such as field margins and riparian buffers can function as havens for natural populations by creating connecting corridors and refuges (3–5). While studies have documented declines in species diversity in agriculture dominated landscapes (6–8), species that persist in these environments may also experience shifts in diversity on the level of individual trait expression resulting in phenotypic clines across land use gradients. Examining intraspecific trait responses to landscape modification may provide further insight into species sensitivity to environmental change and help predict the consequences for ecosystem function (9).

There is growing evidence that trait shifts associated with phenotypic clines may contribute substantially to community trait variation, with significant implications for ecosystem processes (9–11). In plants, intraspecific trait variation has been found to account for 32% of total trait variation among communities (12). This variation can be attributed to a combination of heritable differences as well as plasticity (13) which collectively are an important mechanism allowing plants to track changing environmental conditions (14,15). Studies have found that wild plants experience fitness costs in landscapes dominated by agriculture (16,17) raising questions about wild plants' capacity to track the unprecedented speed of anthropogenic environmental change.

There is already some evidence of phenotypic clines corresponding with human mediated landscape modification. Collectively these studies documented larger floral displays, reduced

defenses, and advanced phenology in plants within highly modified landscapes (18–23). Though these studies found similar patterns of phenotypic change, the predicted mechanism driving this change was not consistent in all studies. For example, both Moreira et al. (2019) and Thompson et al. (2016) documented a reduction in chemical defenses for plants in urbanized landscapes, however, the former was explained by variation in herbivore damage, while the latter was explained by colder ground temperatures in winter. Shifts in floral traits were consistently attributed to pollinator visitation (18,20). While these studies uncover important patterns and drivers of trait change associated with human mediated landscape modification, they are restricted to urbanization gradients, leaving patterns across agricultural gradients largely unexplored (24). Given that agricultural landscapes cover over 40% of the earth surface (1,25), this represents a critical gap in our understanding of plant responses to landscape modification.

Like urban landscapes, agricultural landscapes are characterized by unique biotic and abiotic conditions with the potential to alter trait distributions within local plant populations. For example, agricultural landscapes have been associated with a reduction in the abundance and diversity of pollinating insects (26,27), soil degradation (28), and contamination by agrochemicals (29,30). A phenotypic cline would provide evidence that plants are responding to these novel environmental conditions. It is important to understand trait responses of plants growing in semi-natural spaces within agricultural landscapes due to the critical ecosystem services they provide by supplementing floral resources for pollinators (31,32), creating reservoirs for natural enemies (33,34), and connecting fragmented habitat patches (35,36). Therefore, trait changes have the potential not only to affect plant species persistence in modified landscapes, but also shape the community around them. Plant traits have been found to mediate

intraspecific diversity in insects (37), highlighting the potential for shifts in intraspecific plant traits to have cascading effects on the broader community.

In this study we test for evidence of a phenotypic cline across a gradient of agricultural landscape modification in three short-lived wild Brassicaceae: *Barbarea vulgaris* (Yellow Rocket), *Thlaspi arvense* (Field Pennycress) and *Capsella bursa-pastoris* (Shepherd's Purse). We collected seeds from plants growing in field margins along a gradient of increasing agricultural area in the surrounding landscape (13-16 populations) and grew their progeny together in a greenhouse common garden. We measured reproductive and fitness traits, including flower size and seed set and conducted a caterpillar no-choice bioassay to evaluate defensive traits. Ultimately, this study seeks to examine wild plant responses to agricultural landscape modification, thus providing a foundation for formulating hypotheses and predictions about the mechanisms of landscape mediated trait change in future studies.

Materials and Methods

Study System

While our three study species originate from Eurasia, they have a global distribution and are considered naturalized in North America as weeds (38). These species provide a useful system to study landscape-mediated trait adaptation because they are abundant even in landscapes dominated by agriculture. Because they persist across the entire landscape gradient, these species are exposed to the full range of variation imposed by human-mediated landscape modification. All three species utilize mixed mating systems, but the potential for self-pollination varies by species, with *T. arvense* and *C. bursa-pastoris* producing a high proportion of self-fertilized seeds, while *B. vulgaris* is primarily outcrossing (*personal observation*). All three

species also contain glucosinolate secondary metabolites which function as a defense against generalist herbivores and as a feeding stimulant for specialists.

Landscape Analysis

Parent seeds were collected from sites in the Finger Lakes Region of New York State, USA in the fall 2019. Land use composition at each site was evaluated using the 2019 National Agricultural Statistics Service Cropland Data Layer for New York (USDA 2019) in QGIS 3.16 (QGIS Development Team, QGIS Geographic Information System). Within a 500, 1000, and 1500 m radius of each collection site we calculated the proportion categorized as agriculture, pasture, urban development, natural open and natural forested land area (see Supporting Information Table S1 for detailed land cover classifications). To confirm that the landscape composition has been consistent for at least the last decade, allowing for adaptation to take place across the gradient of landscape modification, we regressed agricultural landscape composition in 2008 against each subsequent year based on the USDA Cropland Data Layer. The strong linear relationship indicates landscape stability during this time (Fig 1; $R^2 > 0.91$, $F = 5136.38$, $p < 0.001$).

Fig 1. Landscape composition has been consistent for the last decade.

Correlation between the USDA Cropland Data Layer landscape classification for agricultural land cover at 1500m in 2008 and each subsequent year through 2018 for each collection site. Each point represents a collection site, and each line represents the landscape composition across all sites in a different year compared to 2008.

Seed Collection from Wild Populations

At each site, parent plants were chosen in semi-natural patches alongside cultivated crops (field margins, fallow fields, ditches). The natural variation in landscape composition at the

broader scale from 500-1500m around each collection site represented a gradient with an increasing proportion of agricultural land cover. Seeds of *B. vulgaris* were collected at 13 sites from at least five parent plants at all sites except one where only four parent plants were present. Seed of *T. arvense* were collected from 15 sites, with at least five parent plants at all but two sites where only three parent plants were present. For *C. bursa-pastoris*, seeds were collected at 16 sites, with at least five parent plants at all sites except two where only four parent plants were present. Seeds were allowed to fully dry on the plant before collection and were kept separated by parent and stored in the freezer for at least a month to promote germination.

Germination and Propagation

For each species, twenty-five seeds from each of five randomly selected parents (or the maximum number of parents available when less than five parents were found at a site) from each collection site were weighed together to the nearest .01mg to test for differences in seed mass from the field. Seeds were then planted in trays with Cornell Mix (see soil preparation in Supporting Information) in a greenhouse at approximately 20° C with a 12-hour photoperiod and biweekly fertilization with Jack's Professional water-soluble fertilizer 21-5-20 as 300 PPM. Total germination was recorded after 14 days. Seeds germinated from all *B. vulgaris* parent plants, while eight out of 71 *T. arvense* parent plants and 35 of 72 *C. bursa-pastoris* parent plants resulted in no germination. Replanting *C. bursa-pastoris* seeds to account for the low germination resulted in 1-25 germinated seedlings per site. Five randomly selected seedlings per parent (or the maximum number germinated when less than five seedlings from one parent germinated) were transplanted into individual pots (4 x 4 x 3.5 in) with Cornell Mix. The seedlings were arranged randomly in the greenhouse to account for any possible variation in light, temperature, or watering.

As biennials, it is common for brassica species to require a cold period to initiate flowering. All but two *C. bursa-pastoris* plants and 72 *T. arvense* plants bolted without vernalization. The remaining 210 *T. arvense* plants and all *B. vulgaris* plants were moved into a cold growth chamber to vernalize. The temperature was set to 4° C with a 10-hour photoperiod (corresponding to median winter day length) and the plants were watered only as needed to prevent wilting. Plants were moved back into the greenhouse after approximately 4 months. After a week, the plants were transferred to larger pots (6 x 6 x 5.5 in) with Cornell Mix to prevent excessive drying. Out of 319 *B. vulgaris* plants, 308 individuals successfully bloomed after vernalization. All but 8 of the 282 individual *T. arvense* and 2 of the 231 *C. bursa-pastoris* plants successfully bloomed.

Floral Traits

For all species, we removed one of the first 5 flowers that opened on a plant. All petals were removed and fixed to a piece of paper with transparent tape. Two anthers were then removed so the remaining anthers and stigma would lay flat when taped. Petal length and width were measured using a microscope at 12.5 magnification and CellSens software (Olympus SZX10 stereo microscope using the digital measurement tool in the cellSens software (Olympus Corp. Tokyo, Japan)) to the nearest hundredth mm. Anther-stigma distance was evaluated by measuring the length of the stamen and pistil and then subtracting the two values (stamen-pistil). The longest petal was used to measure petal length and width. To estimate petal area, we used the formula for an oval (½ length x ½ width x π). We evaluated self-pollination once the plants had finished blooming by recording the proportion of fertile seed pods out of 30 on three stalks (for a total of 90 per plant) when counting from the base. A seed pod was classified as fertile if at

least one seed was developing inside. Plants were spaced in the greenhouse so that no individuals were touching, to prevent any neighboring plants from exchanging pollen.

Plant Harvest/Seed Collection

For *C. bursa-pastoris* and *T. arvense*, once seed pods had dried but not shattered, we collected the first 15 unshattered seed pods from one of the most central stalks. After the seeds were collected, plants were cut back to the soil surface and placed in paper bags. The bags were left in the greenhouse to dry and moved into a drying oven for 48 hours at approximately 38° C. The plants were weighed immediately after they were removed from the drying oven. The seed pods were collected from the plants were weighed separately. For *C. bursa-pastoris*, seeds and seed pods were weighed together due to difficulty separating the small seeds from shattered pod debris. For *T. arvense*, the seeds were separated easily from the seed pods, and therefore only the seeds themselves were weighed. Due to the COVID19 pandemic related campus closures, *B. vulgaris* seeds were collected before the pods were ripe. The green pods were dried in a drying oven for 3 days before the seeds and seed pods were weighed together. This method was validated using a subset of *B. vulgaris* seed pods from a separate experiment confirming that seed pod mass and seed mass are highly correlated (Pearson's $r = 0.90$, $P < 0.001$).

Bioassay

We conducted no choice bioassays using the generalist herbivore, *Trichoplusia ni*. Bioassays were conducted independently for each species following the same protocol. Seeds from the same parent plants used to evaluate reproductive traits were sown in a growth chamber with a temperature of 21° C, 15 h fluorescent light per day and daily watering. Once the seedlings had produced at least four true leaves, we randomly selected and transferred two seedlings from each parent to individual pots (4 x 4 x 3.5 in) with Cornell Mix and randomized

the position of the pots among rows and columns within the growth chamber. Approximately 2-4 weeks after transplanting (depending on the species) two leaves were removed from each plant starting with the first fully expanded leaf and continuing around the rosette. Each leaf was placed in an individual florist tube with water and enclosed in a plastic cup to contain the caterpillar with the leaf. Each leaf received one caterpillar that had fed on Southland Products Cabbage Looper Diet for two days to maximize caterpillar survival. Caterpillars were weighed immediately prior to placement and remained on the leaves for 3 days in a growth chamber with a temperature of 25° C and 15 h of fluorescent light per day. On the third day, we weighed the caterpillars to the nearest .01mg and measured the total leaf area and leaf area consumed using LeafByte (39). The leaf measurements were taken within 3 days of the conclusion of the feeding trial.

Statistics

We used R programming software (v.4.1.0) for statistical analyses (R Core Team 2021). Given the high number of landscape variables and scales (Supporting Information Table S1), we conducted a principal component analysis combining the most predictive landscape types for each species across scales to produce PC1 and PC2 values that summarized the relative position of each source site (Fig 2). The PC1 value represents increasing agricultural land cover, explaining 47.6% of the variation in landscape composition, and was used for all species in all further analyses. We consider these results to be highly conservative given that we found each species showed the strongest response to a unique landscape type and scale when analyzed independently (see Supporting Information Fig S1, S2, and S3; Tables S3, S4, and S5). We present the results from the principal component landscape variable here to uncover generalizable patterns of trait change associated with agriculture.

Fig 2. Principal component analysis summarizing landscape composition across sites. A principal component analysis combining the most predictive landscape types (agriculture, pasture, and unforested natural land covers) for all study species across three scales surrounding the collection site (500, 1000 and 1500m). Each point represents a site where seeds were collected. The first two principal components account for at least 82% of the variation in landscape composition. PC1 has positive and negative loadings reflecting agricultural land cover, and PC2 has positive and negative loadings reflecting pastural land cover.

To evaluate changes in trait expression across the land use gradient we fit separate linear mixed models using the R package lme4 with each trait as the response variable and the PC1 landscape variable described in the previous analysis as a fixed effect. We fit individual models for each species and trait combination (field collected seed mass, germination rate, petal area, anther-stigma distance, plant mass, self-pollination rate, self-pollination seed mass, caterpillar consumption efficiency, leaf area consumed, and caterpillar relative growth rate). Each model included a random effect of parent plant to control for multiple offspring from each parent which was nested within collection site to control for non-independence of parents within a site. Models for the parent-level traits of field collected seed mass and germination rate only included collection site as a random effect. For models evaluating petal size, we included plant mass as a predictor to control for variation in plant size. In each model evaluating *T. arvense* reproductive traits (petal area, seed mass, plant mass) we included vernalization as a random effect. We also ran a logistic regression to test the extent that the landscape gradient explains the probability that a *T. arvense* plant required vernalization to bloom.

To evaluate defensive traits, we used a logistic regression to test if caterpillar survival correlated significantly with the landscape gradient. Given there was low mortality that did not

correlate with parental source landscape, we excluded any caterpillars that did not survive to the end of the experiment in all future models. In the models evaluating defensive traits from the bioassay (caterpillar consumption efficiency, leaf area consumed, and caterpillar relative growth rate), we included bolting status as a random effect for *T. arvense* and *C. bursa-pastoris*. For all models, we evaluated the significance of individual trait responses using a type three ANOVA. Mantel tests indicated spatial autocorrelation in the residuals of the model testing self-pollination rate in *B. vulgaris,* and the site coordinates were therefore included as fixed effects in this model (Dray & Dufour, 2007; Supporting Information Table S2). There was no significant spatial autocorrelation in the residuals for all other models (Supporting Information Table S2).

Results

Field Collected Data

Neither the seed mass nor germination rates of field collected seed correlated significantly with the land use gradient (Table 1) except for C. *bursa-pastoris,* which had lower germination in higher agricultural landscapes ($\chi^2 = 6.7344$, $P < 0.01$, Fig 3, Table 1).

Fig 3. Reduced germination rate in higher agricultural landscapes for *C. bursa-pastoris*.

The proportion *C. bursa-pastoris* seeds germinated out of 25 seeds per parent plant decreases across a gradient of increasing agricultural habitat modification. Higher PC1 values indicate a greater proportion agricultural land cover in the surrounding landscape, while lower values indicate greater natural land cover. Solid regression lines indicate significant correlations ($P < 0.05$), dashed indicate marginal correlations ($P < 0.1$), and no regression line indicates no relationship. Error bars represent standard error.

Table 1. Analysis of landscape effects on seed germination rate for three species.

Species	Response variable	N Parents	N Sites	Predictor	Chisq	Df	p-value
B. vulgaris	Field Seed Mass	63	13	PC1	1.0696	1	0.301

	Germination Rate	63	13	PC1	0.2264	1	0.6342
C. bursa-pastoris	Field Seed Mass	78	16	PC1	0.0085	1	0.9264
	**Germination Rate**	**78**	**16**	**PC1**	**6.7344**	**1**	**0.0095**
T. arvense	Field Seed Mass	71	15	PC1	0.9013	1	0.3424
	Germination Rate	71	15	PC1	0.2911	1	0.5895

Results of individual general linear mixed models investigating the effects of agricultural landscape modification (PC1) on field collected seed mass and germination rate in three wild brassica species. Agricultural landscape modification refers to the extent of agricultural land cover in a radius surrounding the collection site. Collection site was included as a random effect in all models. Statistically significant predictors are indicated in bold ($P < 0.05$).

Reproductive Traits

For both *B. vulgaris* and *C. bursa-pastoris*, petal area decreased with increasing agricultural landscape modification, while *T. arvense* petal size did not change (*B. vulgaris:* χ^2 = 10.53, $P < 0.01$, *C. bursa-pastoris*: χ^2 = 4.34, $P < 0.01$, Fig 4, Table 2). However, for *T. arvense,* plants from landscapes with higher agricultural landscape modification were more likely to require vernalization to bloom than plants from more natural landscapes (χ^2 = 38.08, $P <$ 0.0001).

Fig 4. Increasing agricultural land cover associated with reduced petal area in *B. vulgaris* and *C. bursa-pastoris*

Petal area of *B. vulgaris, C. bursa-pastoris,* and *T. arvense* plants collected as seeds from across a gradient of increasing agricultural land cover and grown in a greenhouse common garden. Higher PC1 values indicate a greater proportion agricultural land cover in the surrounding landscape, while lower values indicate greater natural land cover. Solid regression lines indicate significant correlations ($P < 0.05$), dashed indicate marginal correlations ($P < 0.1$), and no regression line indicates no relationship. Error bars represent standard error.

We found no correlations for any species between stigma-anther distance, plant height, plant mass, or auto-fertility and agricultural landscape modification. In *C. bursa-pastoris*, self-pollinated seed mass declined significantly with increasing agricultural land cover (Table 2, Fig 5). There was a marginal negative correlation for *B. vulgaris* self-pollination rate and a marginal positive correlation for *T. arvense* seed mass with increasing agricultural landscape modification (*B. vulgaris*: χ^2 = 366, P = 0.056, *T. arvense*: χ^2 = 3.46, P = 0.063, Table 2, Fig 5).

Fig 5. Increasing agricultural land cover associated with reduced self-pollinated seed mass in *C. bursa-pastoris.*

Mass of seeds from 15 pods produced by self-pollination in *B. vulgaris*, *C. bursa-pastoris*, and *T. arvense* plants sourced from across a gradient of increasing agricultural land cover and grown in a greenhouse common garden. Higher PC1 values indicate a greater proportion agricultural land cover in the surrounding landscape, while lower values indicate greater natural land cover. Solid regression lines indicate significant correlations ($P < 0.05$), dashed indicate marginal correlations ($P < 0.1$), and no regression line indicates no relationship. Error bars represent standard error.

Table 2. Analysis of landscape affects on plant reproductive traits.

Species	Response variable	N plants	N Sites	Predictor	Chisq	Df	p-value
B. vulgaris	**Petal Area**	**309**	**13**	**PC1**	**10.531**	**1**	**0.0012**
				Plant Mass	0.0093	1	0.9234
	Stigma-Anther Distance	312	13	PC1	0.0006	1	0.9803
	Plant Mass	311	13	PC1	0.1542	1	0.6946
	Self-Pollination Rate	*305*	*13*	*PC1*	*3.6598*	*1*	*0.0557*
				**X Coordinate**	**8.2280**	**1**	**0.0041**
				Y Coordinate	0.3644	1	0.5461
	Self-Pollinated Seed Mass	307	13	PC1	0.9705	1	0.3246
C. bursa-pastoris	**Petal Area**	**196**	**16**	**PC1**	**4.3404**	**1**	**0.0372**
				Plant Mass	*3.0199*	*1*	*0.0823*
	Stigma-Anther Distance	216	16	PC1	1.6765	1	0.1954
	Plant Mass	209	16	PC1	0.9104	1	0.34
	Self-Pollination Rate	218	16	PC1	0.8209	1	0.3649
	**Self-Pollinated Seed Mass**	**201**	**16**	**PC1**	**9.5532**	**1**	**0.0020**
T. arvense	Petal Area	250	14	PC1	1.5116	1	0.2189
				Plant Mass	0.3114	1	0.5768
	Stigma-Anther Distance	256	14	PC1	0.2458	1	0.6201
	Plant Mass	269	14	PC1	0.1555	1	0.6933
	Self-Pollination Rate	269	14	PC1	0.0216	1	0.8831
	Self-Pollinated Seed Mass	*241*	*14*	*PC1*	*3.4603*	*1*	*0.0629*

Results of general linear mixed models investigating the effects of agricultural landscape modification (PC1) on petal size, stigma-anther distance, plant mass, auto-fertility, and mass of self-fertilized seeds in three wild brassica species. Agricultural landscape modification refers to the extent of agricultural land cover in a radius surrounding the collection site. Collection site and parent plant were included as a random effect in all models. The site coordinates are included in the model of self-pollination rate in *B. vulgaris* to account for spatial autocorrelation in the residuals of the initial model. For *T. arvense*, vernalization status was also included as a random effect to account for the proportion of plants that underwent a cold treatment to initiate

bloom. Statistically significant predictors ($P < 0.05$) are indicated in bold and marginal predictors ($P < 0.1$) are italicized.

Defense Assessment

In the herbivore no-choice bioassay, 96.8% of 252 *T. ni* caterpillars survived on *B. vulgaris*, 88.7% survived out of 248 on *T. arvense*, and 97.3% survived out of 300 on *C. bursa-pastoris*. The likelihood of caterpillar mortality did not correlate with increasing agricultural land cover (*B. vulgaris:* $\chi^2 = 0.135$, $P = 0.713$; *C. bursa-pastoris*: $\chi^2 = 0.273$, $P = 0.601$; *T. arvense:* $\chi^2 = 1.229$, $P = 0.268$). For both *B. vulgaris* and *C. bursa-pastoris*, caterpillars consumed less leaf area on plants from landscapes with greater agricultural land cover (Table 3, Fig 6). For all species, the leaf area (a proxy for leaf age) and *T. ni* initial mass were positively correlated with consumed leaf area (Table 3). Leaf area was positively correlated with caterpillar relative growth rate for all species, while agricultural land cover had no effect (Table 3). Caterpillar consumption efficiency was significantly positively correlated with agricultural land cover in *C. bursa-pastoris*, but not in *B. vulgaris* and *T. arvense* (Table 3, Fig 7). Caterpillar initial mass was a significant positive predictor of consumption efficiency for *B. vulgaris* and negative predictor for *T. arvense*. Leaf area was a significant negative predictor of consumption efficiency for *C. bursa-pastoris*.

Fig 6. Higher agricultural land cover associated with reduced leaf consumption

Leaf area consumed by *T. ni* caterpillars in a no-choice bioassay feeding on *B. vulgaris, C. bursa-pastoris,* and *T. arvense* plants collected as seeds from across a gradient of increasing agricultural land cover and grown in a greenhouse common garden. Higher PC1 values indicate a greater proportion agricultural land cover in the surrounding landscape, while lower values

indicate greater natural land cover. Error bars represent standard error and solid regression lines indicate significant correlations ($P < 0.05$).

Fig 7. Higher agricultural land cover associated with increased herbivore consumption efficiency

Caterpillar consumption efficiency (mass gained per unit area leaf consumed) in a no-choice bioassay feeding on *B. vulgaris, C. bursa-pastoris,* and *T. arvense* plants collected as seeds from across a gradient of increasing agricultural land cover and grown in a greenhouse common garden. Higher PC1 values indicate a greater proportion agricultural land cover in the surrounding landscape, while lower values indicate greater natural land cover. Error bars represent standard error and solid regression lines indicate significant correlations ($P < 0.05$).

Table 3. Landscape affects on plant defensive traits.

Species	Response variable	N Plants	N Sites	Predictor	Chisq	Df	p-value
B. vulgaris	**Leaf Area Consumed**	**243**	**13**	**PC1**	**6.8196**	**1**	**0.00902**
				**Leaf Size**	**6.9180**	**1**	**0.00853**
				***T. ni* Initial Mass**	**57.461**	**1**	**<0.0001**
	Relative Growth Rate	243	13	PC1	2.6212	1	0.10544
				**Leaf Size**	**3.8853**	**1**	**0.04871**
	Consumption Efficiency	243	13	PC1	2.3731	1	0.12344
				Leaf Size	*2.9017*	*1*	*0.08848*
				***T. ni* Initial Mass**	**5.4733**	**1**	**0.01931**
C. bursa-pastoris	**Leaf Area Consumed**	**288**	**16**	**PC1**	**5.2980**	**1**	**0.02135**
				**Leaf Size**	**28.020**	**1**	**<0.0001**
				***T. ni* Initial Mass**	**30.515**	**1**	**<0.0001**
	Relative Growth Rate	288	16	PC1	0.0841	1	0.77175
				**Leaf Size**	**6.8043**	**1**	**0.00909**
	**Consumption Efficiency**	**288**	**16**	**PC1**	**3.9268**	**1**	**0.04752**
				**Leaf Size**	**19.321**	**1**	**<0.0001**
				T. ni Initial Mass	2.6932	1	0.10078
T. arvense	Leaf Area Consumed	219	13	PC1	0.1570	1	0.69192
				**Leaf Size**	**77.594**	**1**	**<0.0001**
				***T. ni* Initial Mass**	**4.2802**	**1**	**0.03856**
	Relative Growth Rate	216	13	PC1	1.1599	1	0.2815
				**Leaf Size**	**25.276**	**1**	**<0.0001**
	Consumption Efficiency	217	13	PC1	1.8038	1	0.17925
				Leaf Size	0.5398	1	0.46252
				***T. ni* Initial Mass**	**4.7966**	**1**	**0.02852**

Results of general linear mixed models investigating the effects of agricultural landscape modification (PC1) on caterpillar leaf area consumption, relative growth rate, and consumption efficiency (mass gained per unit area eaten) in a no choice bioassay with a generalist herbivore (*Trichoplusia ni*) feeding on three wild brassica species. Agricultural landscape modification refers to the extent of agricultural land cover in a radius surrounding the collection site. Collection site and parent plant were included as a random effect in all models. For *C. bursa-pastoris* and *T. arvense,* bolting status was also included as a random effect as a significant

proportion of the plants began to bloom at the onset of the experiment. Statistically significant predictors ($P < 0.05$) are indicated in bold and marginal predictors ($P < 0.1$) are italicized.

Discussion

The consequences of human mediated landscape modification on biodiversity and ecosystem function are at the forefront of ecological research (41–43). However, the cascading effects of this landscape modification on individual trait responses have, until now, been largely unexplored. Recently, Mitchell et al. (2021) found that proximity to cultivated sunflowers resulted in homogenized selection on floral traits for wild sunflowers. Here, we build on these findings by documenting phenotypic clines in both defensive and reproductive traits across three related plant species. These landscape mediated changes in plant phenotypes have the potential to shape the trait diversity of the broader community (37).

Our findings document a reduction in floral display size in plants from agriculture dominant landscapes for two of the three species examined. This pattern is counter to the findings along urbanization gradients documenting increased floral display size associated with urban environments (18,20), highlighting the unique conditions imposed by each landscapes type. Mitchell et al. (2021) found evidence of selection for higher ray length in wild sunflowers further from cultivated sunflower, aligning with our findings here in Brassicaceae. There are a number of potential biotic and abiotic mechanisms that may be mediating these changes in floral traits (45), however, insect pollinators are frequently identified as key drivers. For example, Brys and Jacquemyn (2012) found that pollinator depleted environments are associated with reduced flower size and increased autonomous self-pollination. There is strong evidence that agricultural landscapes support a reduced abundance and diversity of pollinators (26), making this mechanism a priority for exploration in future studies. A lack of response by the third species (*T.*

arvense) may indicate resilience or that this species is responding by varying another trait not measured in this study such as floral rewards or number of open flowers.

We also found correlations between metrics of plant defense and the land use gradient for two species. *T. ni* caterpillars consumed less leaf area and developed more efficiently on plants from agriculturally dominant landscapes. Arthropod food consumption and utilization is dictated largely by the nutritional quality of the plant and plant defenses, with herbivores consuming more food to compensate for poor diet quality and growing more slowly when a plant expresses higher defenses (47). Given that all plants were grown under equivalent conditions, these observed differences in consumption and assimilation are likely due to plant defenses, but further work is necessary to confirm this mechanism. There is a large body of evidence that herbivore pressure and plant defenses vary predictably along environmental gradients such as elevation, latitude, or plant diversity (48–51). Studies examining plant defenses in urban landscapes have found similar results to those presented here, demonstrating a decrease in plant defenses within modified urban landscapes (21,52). The few studies that have tested these patterns of herbivory in wild plants growing alongside agriculture have shown mixed results, where some report comparable results indicating reduced herbivore pressure associated with agriculture (53), while others document contradictory results of augmented herbivore damage on wild plants (54). There are a number of possible factors contributing to these varied results, including the natural history of the major insect herbivore, the relative attractiveness of adjacent crops compared to the wild plant community, potential insecticide spillover onto wild plants in intensively managed landscapes, and other abiotic factors like temperature (23,55–58).

We examined metrics of fitness through seed mass and germination rate in the wild parent plants and through seed mass and self-fertilization rate in the common garden plants. Of

the field collected data, only *C. bursa-pastoris* demonstrated a significant decline in seed germination in agriculture dominant landscapes. This is consistent with existing studies comparing wild plant reproduction across agricultural gradients (16,59) and may also be the result of a dormancy mechanism adaptive in high disturbance agricultural environments (60). Of the common garden plants, again *C. bursa-pastoris* was the only species that expressed a fitness cost with plants from agriculture dominant landscapes producing lighter seeds through self-fertilization. The combination of low germination rate in seeds from the parent plants and low seed weight in the common garden seeds may indicate inbreeding depression (61), potentially due to population fragmentation associated with homogenized landscapes and reduced pollinator availability to connect isolated populations. The absence of a fitness cost for the other two species could provide evidence of adaptation to the local environment in agricultural landscapes.

In this study we document phenotypic clines across a landscape modification gradient, however, with these data we cannot disentangle the relative degree that plant phenotypic differences can be explained by genetic differences, plasticity, and maternal effects. Given the importance of plasticity and maternal effects in enabling plants to track rapidly changing environmental conditions (62), examining overall trait responses provides a more complete estimate of wild plant responses to agricultural landscape modification. Future studies should test the relative contribution of plasticity and heritable change and examine whether these changes in trait values are adaptive. These studies should evaluate how different plant phenotypes perform across the landscape to test for evidence of selection and document insect interactions and abiotic conditions to identify the mechanisms driving plant trait change.

To date, much of the literature examining the consequences of agricultural landscape modification on plant communities has focused primarily on changes in species diversity. Here

we demonstrate the potential for intraspecific trait change in species that persist within highly modified landscapes, highlighting the need to incorporate trait-based analyses into landscape scale studies. Given that wild plant communities play a critical role in habitat connectivity and stability in highly fragmented, human altered landscapes, understanding how they respond to land use change can inform predictions about the responses and resilience of entire communities (37,63). We hope this work will spur further research examining plant trait change across species and land use types to expose broader patterns of plant trait change in human modified landscapes.

Statements & Declarations

Acknowledgments

We thank Anurag Agrawal, Monica Geber, and Andre Kessler for suggestions regarding project design and comments on earlier drafts of the manuscript. We thank Annika Salzberg, Casey Hale, and Emma Harte for assistance with plant care and data collection and Erika Mudrak at the Cornell Statistical Consulting Center for statistical advice.

Author Contributions

HG and KP conceived the idea for the project and HG conducted preliminary experimental work. HS conducted all experiments reported in the manuscript, analyzed the data, and drafted the manuscript to which HG and KP provided revisions and feedback.

Data Availability Statement

The datasets presented in this study are available in the dryad data repository (DOI https://doi.org/10.5061/dryad.905qfttq6).

Supporting Information Captions

Table S1. CDL land cover types included within the broad land use classifications used in the PCA in the main text and individual analyses included in the supplement.

Table S2. Results of Mantel test of spatial autocorrelation in the residuals of the final models. Statistically significant predictors ($P < 0.05$) are indicated in bold.

Figure S1. Germination rate (A) and seed mass (B) of seeds collected from *B. vulgaris* plants growing at sites representing a gradient of increasing open (non-forested) natural land cover in a 1000m radius around the collection site. Petal area (C), plant mass (D), self-pollinated seed mass (E), percent aborted seed pods (F), and stigma-anther distance (G) of the offspring grown from the parent populations in a greenhouse common garden. Caterpillar consumed leaf area (H) and consumption efficiency (I) from a no-choice herbivore bioassay represent a proxy for plant defense traits in the offspring grown from the parent populations. Solid regression lines indicate significant correlations, dashed indicate marginal correlations, and no regression line indicates no relationship. Error bars represent standard error.

Table S3. Results of general linear mixed models investigating the effects of open natural land cover on all measured traits for *B. vulgaris*. Parent plant nested within collection site was included as a random effect in all models. Statistically significant predictors ($P < 0.05$) are indicated in bold and marginal predictors ($P < 0.1$) are italicized. *Trichoplusia ni* caterpillars used in the leaf bioassay are included in the predictor column as *T.ni*.

Figure S2. Germination rate (A) and seed mass (B) of seeds collected from *C. bursa-pastoris* plants growing at sites representing a gradient of increasing agricultural land cover in a 1500m radius around the collection site. Petal area (C), plant mass (D), self-pollinated seed mass (E), percent aborted seed pods (F), and stigma-anther distance (G) of the offspring grown from the parent populations in a greenhouse common garden. Caterpillar consumed leaf area (H) and

consumption efficiency (I) from a no-choice herbivore bioassay represent a proxy for plant defense traits in the offspring grown from the parent populations. Solid regression lines indicate significant correlations, dashed indicate marginal correlations, and no regression line indicates no relationship. Error bars represent standard error.

Table S4. Results of general linear mixed models investigating the effects of agricultural land cover on all measured traits for *C. bursa-pastoris*. Parent plant nested within collection site was included as a random effect in all models. Statistically significant predictors ($P < 0.05$) are indicated in bold and marginal predictors ($P < 0.1$) are italicized. *Trichoplusia ni* caterpillars used in the leaf bioassay are included in the predictor column as *T.ni*.

Figure S3. Germination rate (A) and seed mass (B) of seeds collected from *T. arvense* plants growing at sites representing a gradient of increasing pasture land cover in a 500m radius around the collection site. Petal area (C), plant mass (D), self-pollinated seed mass (E), percent aborted seed pods (F), and stigma-anther distance (G) of the offspring grown from the parent populations in a greenhouse common garden. Caterpillar consumed leaf area (H) and consumption efficiency (I) from a no-choice herbivore bioassay represent a proxy for plant defense traits in the offspring grown from the parent populations. Solid regression lines indicate significant correlations, dashed indicate marginal correlations, and no regression line indicates no relationship. Error bars represent standard error.

Table S5. Results of general linear mixed models investigating the effects of pasture land cover on all measured traits for *C. bursa-pastoris*. Parent plant nested within collection site was included as a random effect in all models. Statistically significant predictors ($P < 0.05$) are indicated in bold and marginal predictors ($P < 0.1$) are italicized. *Trichoplusia ni* caterpillars used in the leaf bioassay are included in the predictor column as *T.ni*.

References

1. Foley JA. Global Consequences of Land Use. Science. 2005 Jul 22;309(5734):570–4.

2. Watson JEM, Shanahan DF, Di Marco M, Allan J, Laurance WF, Sanderson EW, et al. Catastrophic Declines in Wilderness Areas Undermine Global Environment Targets. Curr Biol. 2016 Nov 7;26(21):2929–34.

3. Hietala-Koivu R, Järvenpää T, Helenius J. Value of semi-natural areas as biodiversity indicators in agricultural landscapes. Agric Ecosyst Environ. 2004 Jan 1;101(1):9–19.

4. Garratt MPD, Senapathi D, Coston DJ, Mortimer SR, Potts SG. The benefits of hedgerows for pollinators and natural enemies depends on hedge quality and landscape context. Agric Ecosyst Environ. 2017 Sep 1;247:363–70.

5. Holland JM, Douma JC, Crowley L, James L, Kor L, Stevenson DRW, et al. Semi-natural habitats support biological control, pollination and soil conservation in Europe. A review. Agron Sustain Dev. 2017 Jul 24;37(4):31.

6. Carmona CP, Guerrero I, Peco B, Morales MB, Oñate JJ, Pärt T, et al. Agriculture intensification reduces plant taxonomic and functional diversity across European arable systems. Funct Ecol. 2020;34(7):1448–60.

7. Roschewitz I, Gabriel D, Tscharntke T, Thies C. The effects of landscape complexity on arable weed species diversity in organic and conventional farming. J Appl Ecol. 2005;42(5):873–82.

8. Tarifa R, Martínez-Núñez C, Valera F, González-Varo JP, Salido T, Rey PJ. Agricultural intensification erodes taxonomic and functional diversity in Mediterranean olive groves by filtering out rare species. J Appl Ecol. 2021;58(10):2266–76.

9. Liu C, Li Y, Yan P, He N. How to Improve the Predictions of Plant Functional Traits on Ecosystem Functioning? Front Plant Sci [Internet]. 2021 [cited 2022 Aug 15];12. Available from: https://www.frontiersin.org/articles/10.3389/fpls.2021.622260

10. Fu H, Yuan G, Jeppesen E. Trait-based community assembly of submersed macrophytes subjected to nutrient enrichment in freshwater lakes: Do traits at the individual level matter? Ecol Indic. 2020 Mar 1;110:105895.

11. Malyshev AV, Arfin Khan MAS, Beierkuhnlein C, Steinbauer MJ, Henry HAL, Jentsch A, et al. Plant responses to climatic extremes: within-species variation equals among-species variation. Glob Change Biol. 2016;22(1):449–64.

12. Siefert A, Violle C, Chalmandrier L, Albert CH, Taudiere A, Fajardo A, et al. A global meta-analysis of the relative extent of intraspecific trait variation in plant communities. Ecol Lett. 2015;18(12):1406–19.

13. Matesanz S, Horgan-Kobelski T, Sultan SE. Phenotypic Plasticity and Population Differentiation in an Ongoing Species Invasion. PLOS ONE. 2012 Sep 19;7(9):e44955.

14. Björklund M, Ranta E, Kaitala V, Bach LA, Lundberg P, Stenseth NC. Quantitative Trait Evolution and Environmental Change. PLOS ONE. 2009 Feb 20;4(2):e4521.

15. Norberg J, Swaney DP, Dushoff J, Lin J, Casagrandi R, Levin SA. Phenotypic diversity and ecosystem functioning in changing environments: A theoretical framework. Proc Natl Acad Sci. 2001 Sep 25;98(20):11376–81.

16. Holzschuh A, Dormann CF, Tscharntke T, Steffan-Dewenter I. Expansion of mass-flowering crops leads to transient pollinator dilution and reduced wild plant pollination. Proc R Soc B Biol Sci. 2011 Nov 22;278(1723):3444–51.

17. Van Reeth C, Michel N, Bockstaller C, Caro G. Influences of oilseed rape area and aggregation on pollinator abundance and reproductive success of a co-flowering wild plant. Agric Ecosyst Environ. 2019 Aug 1;280:35–42.

18. Bode RF, Tong R. Pollinators exert positive selection on flower size on urban, but not on rural Scotch broom (Cytisus scoparius L. Link). J Plant Ecol. 2018 Mar 6;11(3):493–501.

19. Fisogni A, Hautekèete N, Piquot Y, Brun M, Vanappelghem C, Michez D, et al. Urbanization drives an early spring for plants but not for pollinators. Oikos. 2020;129(11):1681–91.

20. Irwin RE, Warren PS, Adler LS. Phenotypic selection on floral traits in an urban landscape. Proc R Soc B Biol Sci. 2018 Aug 15;285(1884):20181239.

21. Moreira X, Abdala-Roberts L, Berny Mier y Teran JC, Covelo F, de la Mata R, Francisco M, et al. Impacts of urbanization on insect herbivory and plant defences in oak trees. Oikos. 2019;128(1):113–23.

22. Santangelo JS, Rivkin LR, Advenard C, Thompson KA. Multivariate phenotypic divergence along an urbanization gradient. Biol Lett. 2020 Sep 30;16(9):20200511.

23. Thompson KA, Renaudin M, Johnson MTJ. Urbanization drives the evolution of parallel clines in plant populations. Proc R Soc B Biol Sci. 2016 Dec 28;283(1845):20162180.

24. Schroeder H, Grab H, Kessler A, Poveda K. Human-Mediated Land Use Change Drives Intraspecific Plant Trait Variation. Front Plant Sci [Internet]. 2021 [cited 2021 Jan 22];11. Available from: https://www.frontiersin.org/articles/10.3389/fpls.2020.592881/full?&utm_source=Email_to_authors_&utm_medium=Email&utm_content=T1_11.5e1_author&utm_campaign=Email_publication&field=&journalName=Frontiers_in_Plant_Science&id=592881

25. Ellis EC, Ramankutty N. Putting people in the map: anthropogenic biomes of the world. Front Ecol Environ. 2008;6(8):439–47.

26. Grab H, Branstetter MG, Amon N, Urban-Mead KR, Park MG, Gibbs J, et al. Agriculturally dominated landscapes reduce bee phylogenetic diversity and pollination services. Science. 2019 Jan 18;363(6424):282–4.

27. Mallinger RE, Gibbs J, Gratton C. Diverse landscapes have a higher abundance and species richness of spring wild bees by providing complementary floral resources over bees' foraging periods. Landsc Ecol. 2016 Sep 1;31(7):1523–35.

28. Baude M, Meyer BC, Schindewolf M. Land use change in an agricultural landscape causing degradation of soil based ecosystem services. Sci Total Environ. 2019 Apr 1;659:1526–36.

29. Russo L, Buckley YM, Hamilton H, Kavanagh M, Stout JC. Low concentrations of fertilizer and herbicide alter plant growth and interactions with flower-visiting insects. Agric Ecosyst Environ. 2020 Dec 1;304:107141.

30. Ward LT, Hladik ML, Guzman A, Winsemius S, Bautista A, Kremen C, et al. Pesticide exposure of wild bees and honey bees foraging from field border flowers in intensively managed agriculture areas. Sci Total Environ. 2022 Jul 20;831:154697.

31. Beduschi T, Kormann UG, Tscharntke T, Scherber C. Spatial community turnover of pollinators is relaxed by semi-natural habitats, but not by mass-flowering crops in agricultural landscapes. Biol Conserv. 2018 May 1;221:59–66.

32. Raderschall CA, Bommarco R, Lindström SAM, Lundin O. Landscape crop diversity and semi-natural habitat affect crop pollinators, pollination benefit and yield. Agric Ecosyst Environ. 2021 Feb 1;306:107189.

33. Pollier A, Tricault Y, Plantegenest M, Bischoff A. Sowing of margin strips rich in floral resources improves herbivore control in adjacent crop fields. Agric For Entomol. 2019;21(1):119–29.

34. Arnold SEJ, Elisante F, Mkenda PA, Tembo YLB, Ndakidemi PA, Gurr GM, et al. Beneficial insects are associated with botanically rich margins with trees on small farms. Sci Rep. 2021 Jul 26;11(1):15190.

35. Holzschuh A, Steffan-Dewenter I, Tscharntke T. Grass strip corridors in agricultural landscapes enhance nest-site colonization by solitary wasps. Ecol Appl. 2009;19(1):123–32.

36. Delattre T, Pichancourt JB, Burel F, Kindlmann P. Grassy field margins as potential corridors for butterflies in agricultural landscapes: A simulation study. Ecol Model. 2010 Jan 24;221(2):370–7.

37. Grass I, Albrecht J, Farwig N, Jauker F. Plant traits and landscape simplification drive intraspecific trait diversity of Bombus terrestris in wildflower plantings. Basic Appl Ecol. 2021 Dec 1;57:91–101.

38. MacDonald MA, Cavers PB. The biology of Canadian weeds.: 97. *Barbarea vulgaris* R.Br. Can J Plant Sci. 1991 Jan 1;71(1):149–66.

39. Getman-Pickering ZL, Campbell A, Aflitto N, Grele A, Davis JK, Ugine TA. LeafByte: A mobile application that measures leaf area and herbivory quickly and accurately. Methods Ecol Evol. 2020;11(2):215–21.

40. Dray S, Dufour AB. The ade4 Package: Implementing the Duality Diagram for Ecologists. J Stat Softw. 2007 Sep 30;22:1–20.

41. Dainese M, Martin EA, Aizen MA, Albrecht M, Bartomeus I, Bommarco R, et al. A global synthesis reveals biodiversity-mediated benefits for crop production. Sci Adv. 2019 Oct 1;5(10):eaax0121.

42. Gámez-Virués S, Perović DJ, Gossner MM, Börschig C, Blüthgen N, de Jong H, et al. Landscape simplification filters species traits and drives biotic homogenization. Nat Commun. 2015 Oct 20;6(1):1–8.

43. Kremen C, Miles A. Ecosystem Services in Biologically Diversified versus Conventional Farming Systems: Benefits, Externalities, and Trade-Offs. Ecol Soc [Internet]. 2012 [cited 2020 Dec 10];17(4). Available from: https://www.jstor.org/stable/26269237

44. Mitchell N, Chamberlain SA, Whitney KD. Proximity to crop relatives determines some patterns of natural selection in a wild sunflower. Evol Appl. 2021;14(5):1328–42.

45. Caruso CM, Eisen KE, Martin RA, Sletvold N. A meta-analysis of the agents of selection on floral traits. Evolution. 2019;73(1):4–14.

46. Brys R, Jacquemyn H. Effects of human-mediated pollinator impoverishment on floral traits and mating patterns in a short-lived herb: an experimental approach. Funct Ecol. 2012;26(1):189–97.

47. Couture JJ, Mason CJ, Habeck CW, Lindroth RL. Behavioral and morphological responses of an insect herbivore to low nutrient quality are inhibited by plant chemical defenses. Arthropod-Plant Interact. 2016 Aug 1;10(4):341–9.

48. Schuldt A, Baruffol M, Böhnke M, Bruelheide H, Härdtle W, Lang AC, et al. Tree diversity promotes insect herbivory in subtropical forests of south-east China. J Ecol. 2010;98(4):917–26.

49. Callis-Duehl K, Vittoz P, Defossez E, Rasmann S. Community-level relaxation of plant defenses against herbivores at high elevation. Plant Ecol. 2017 Mar;218(3):291–304.

50. Bakhtiari M, Formenti L, Caggìa V, Glauser G, Rasmann S. Variable effects on growth and defense traits for plant ecotypic differentiation and phenotypic plasticity along elevation gradients. Ecol Evol. 2019;9(7):3740–55.

51. Hargreaves AL, Suárez E, Mehltreter K, Myers-Smith I, Vanderplank SE, Slinn HL, et al. Seed predation increases from the Arctic to the Equator and from high to low elevations. Sci Adv [Internet]. 2019 Feb [cited 2022 Jan 7]; Available from: https://www.science.org/doi/abs/10.1126/sciadv.aau4403

52. Santangelo JS, Ness RW, Cohan B, Fitzpatrick CR, Innes SG, Koch S, et al. Global urban environmental change drives adaptation in white clover. Science. 2022 Mar 18;375(6586):1275–81.

53. Chamberlain SA, Whitney KD, Rudgers JA. Proximity to agriculture alters abundance and community composition of wild sunflower mutualists and antagonists. Ecosphere. 2013;4(8):art96.

54. McKone MJ, McLauchlan KK, Lebrun EG, McCall AC. An Edge Effect Caused by Adult Corn-Rootworm Beetles on Sunflowers in Tallgrass Prairie Remnants. Conserv Biol. 2001;15(5):1315–24.

55. Blitzer EJ, Dormann CF, Holzschuh A, Klein AM, Rand TA, Tscharntke T. Spillover of functionally important organisms between managed and natural habitats. Agric Ecosyst Environ. 2012 Jan 1;146(1):34–43.

56. Botías C, David A, Hill EM, Goulson D. Contamination of wild plants near neonicotinoid seed-treated crops, and implications for non-target insects. Sci Total Environ. 2016 Oct 1;566–567:269–78.

57. Dong Z, Zhang Q, Li L, Lu Z, Li C, Ouyang F, et al. Landscape agricultural simplification correlates positively with the spatial distribution of a specialist yet negatively with a generalist pest. Sci Rep. 2020 Jan 15;10(1):344.

58. Whitehead SR, Turcotte MM, Poveda K. Domestication impacts on plant–herbivore interactions: a meta-analysis. Philos Trans R Soc B Biol Sci. 2017 Jan 19;372(1712):20160034.

59. Chateil C, Porcher E. Landscape features are a better correlate of wild plant pollination than agricultural practices in an intensive cropping system. Agric Ecosyst Environ. 2015 Mar 1;201:51–7.

60. Toorop PE, Campos Cuerva R, Begg GS, Locardi B, Squire GR, Iannetta PPM. Co-adaptation of seed dormancy and flowering time in the arable weed Capsella bursa-pastoris (shepherd's purse). Ann Bot. 2012 Feb 1;109(2):481–9.

61. Delmas CE, Cheptou PO, Escaravage N, Pornon A. High lifetime inbreeding depression counteracts the reproductive assurance benefit of selfing in a mass-flowering shrub. BMC Evol Biol. 2014 Nov 30;14(1):243.

62. Galloway LF. Maternal effects provide phenotypic adaptation to local environmental conditions. New Phytol. 2005;166(1):93–100.

63. Schmitz OJ, Buchkowski RW, Burghardt KT, Donihue CM. Chapter Ten - Functional Traits and Trait-Mediated Interactions: Connecting Community-Level Interactions with Ecosystem Functioning. In: Pawar S, Woodward G, Dell AI, editors. Advances in Ecological Research [Internet]. Academic Press; 2015 [cited 2020 Dec 10]. p. 319–43. (Trait-Based Ecology - From Structure to Function; vol. 52). Available from: http://www.sciencedirect.com/science/article/pii/S0065250415000045

Figure 1

Click here to access/download;Figure;Fig1.tif

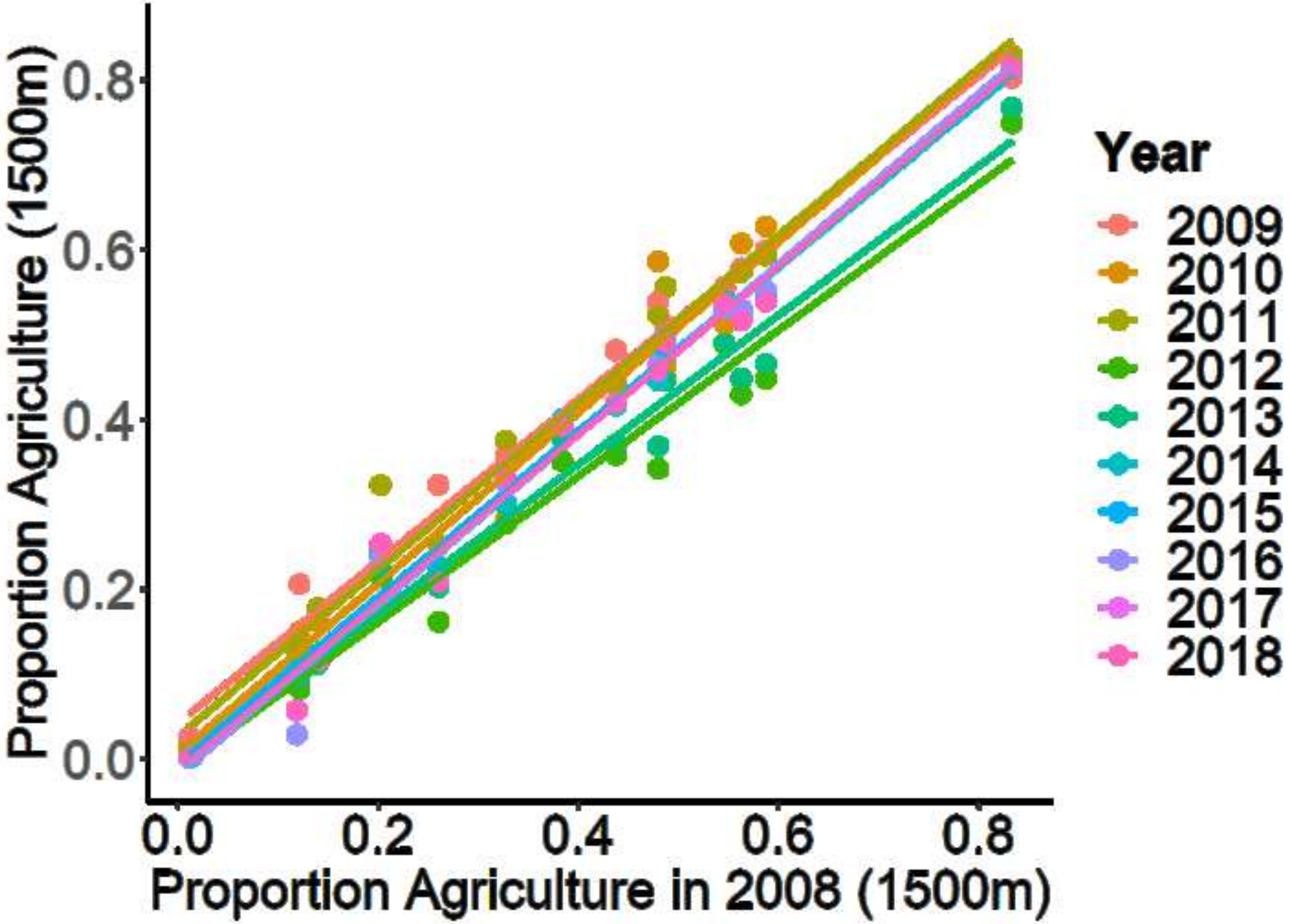

Figure 2 Click here to access/download;Figure;Fig2.jpg

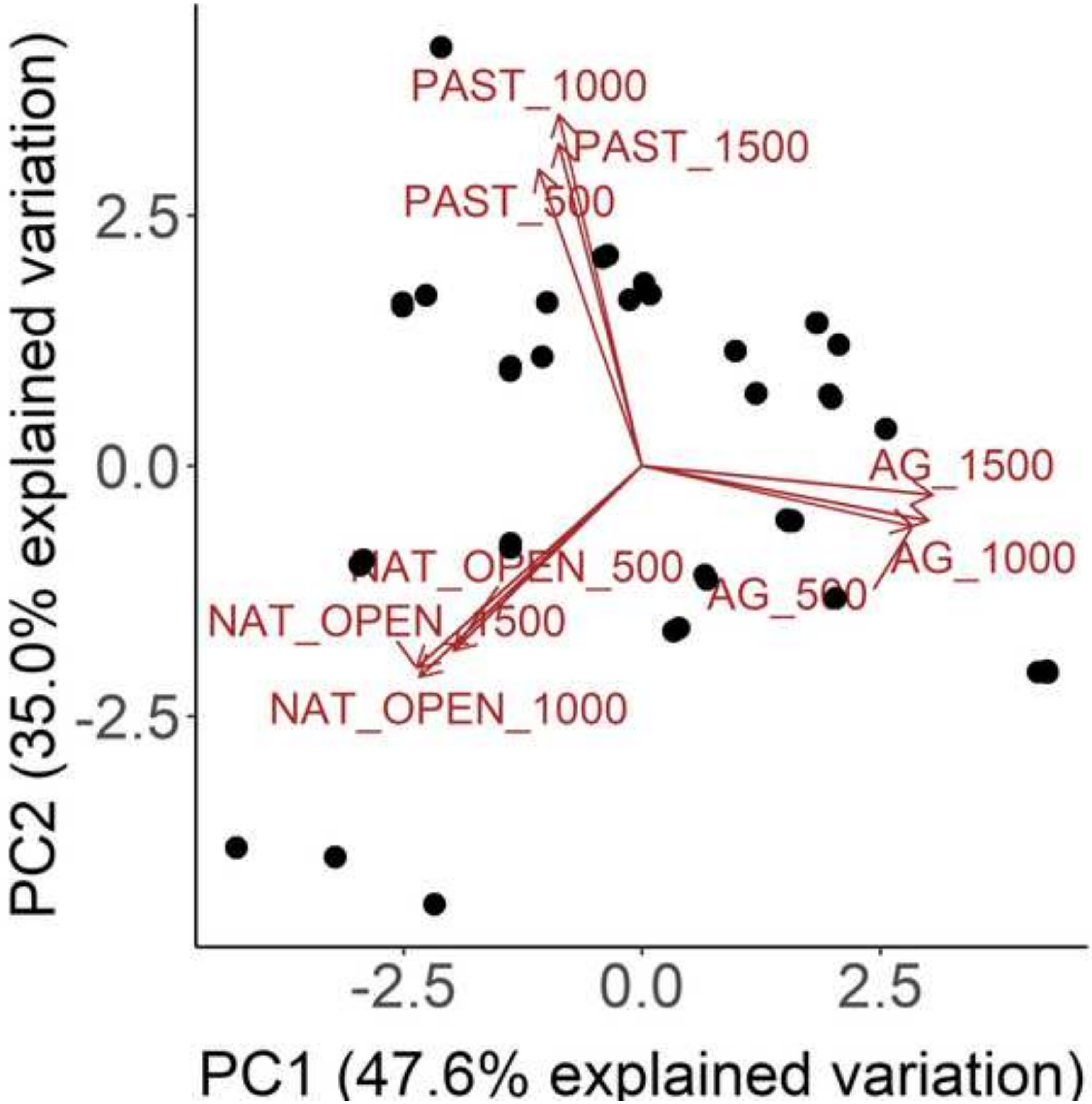

Click here to access/download;Figure;Fig3.tif

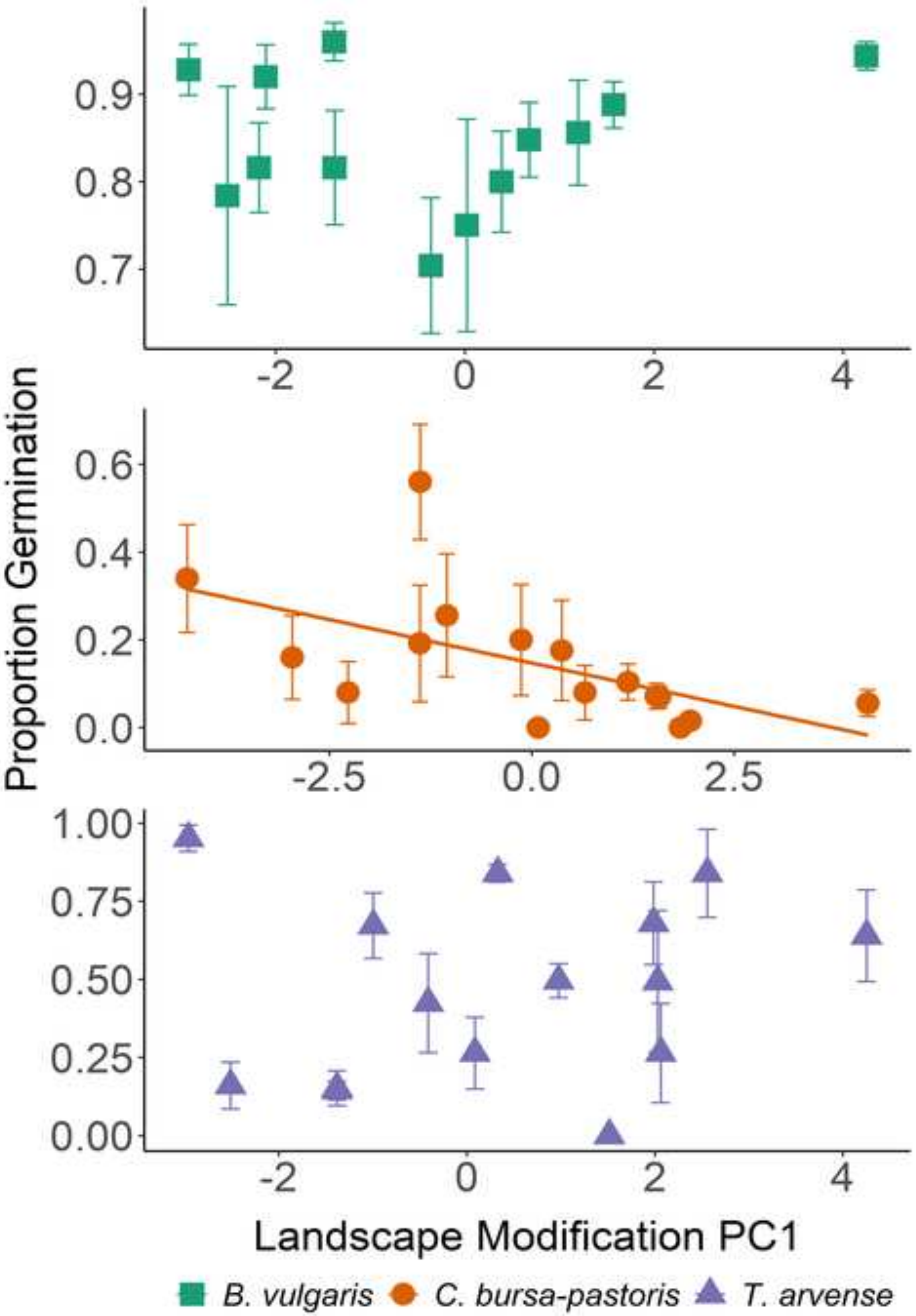

Click here to access/download;Figure;Fig4.tif

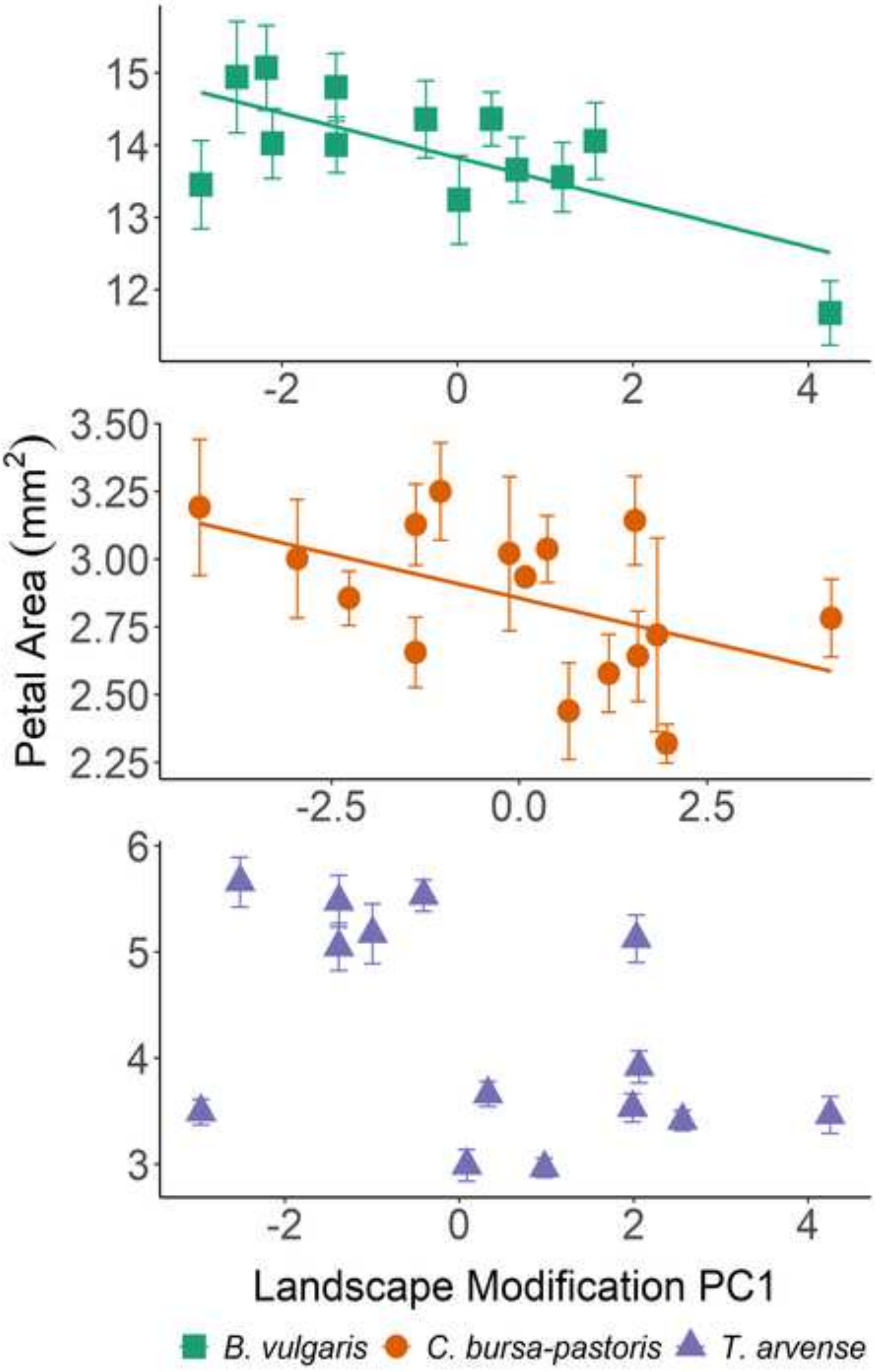

Click here to access/download;Figure;Fig5.tif

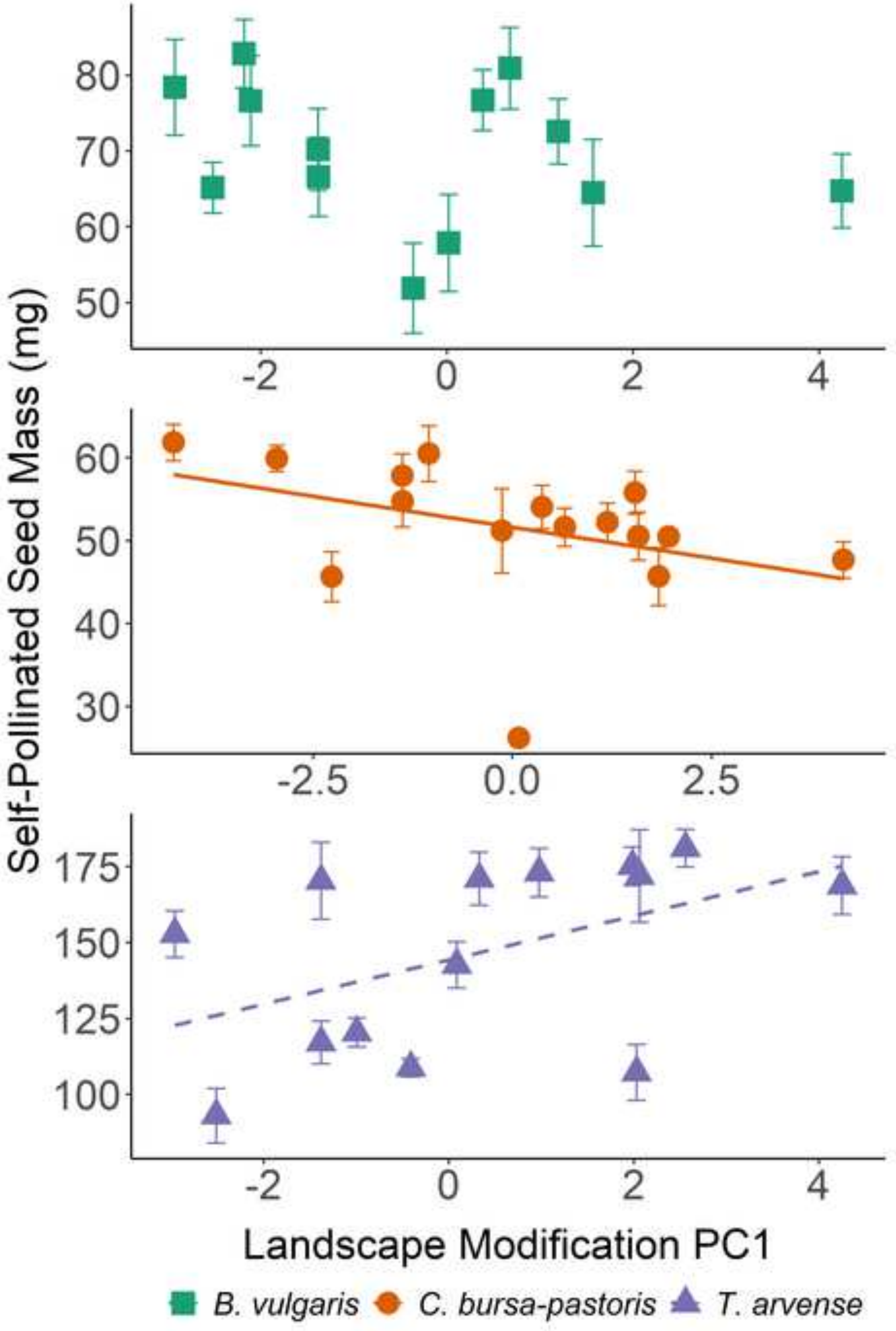

Click here to access/download;Figure;Fig6.tif

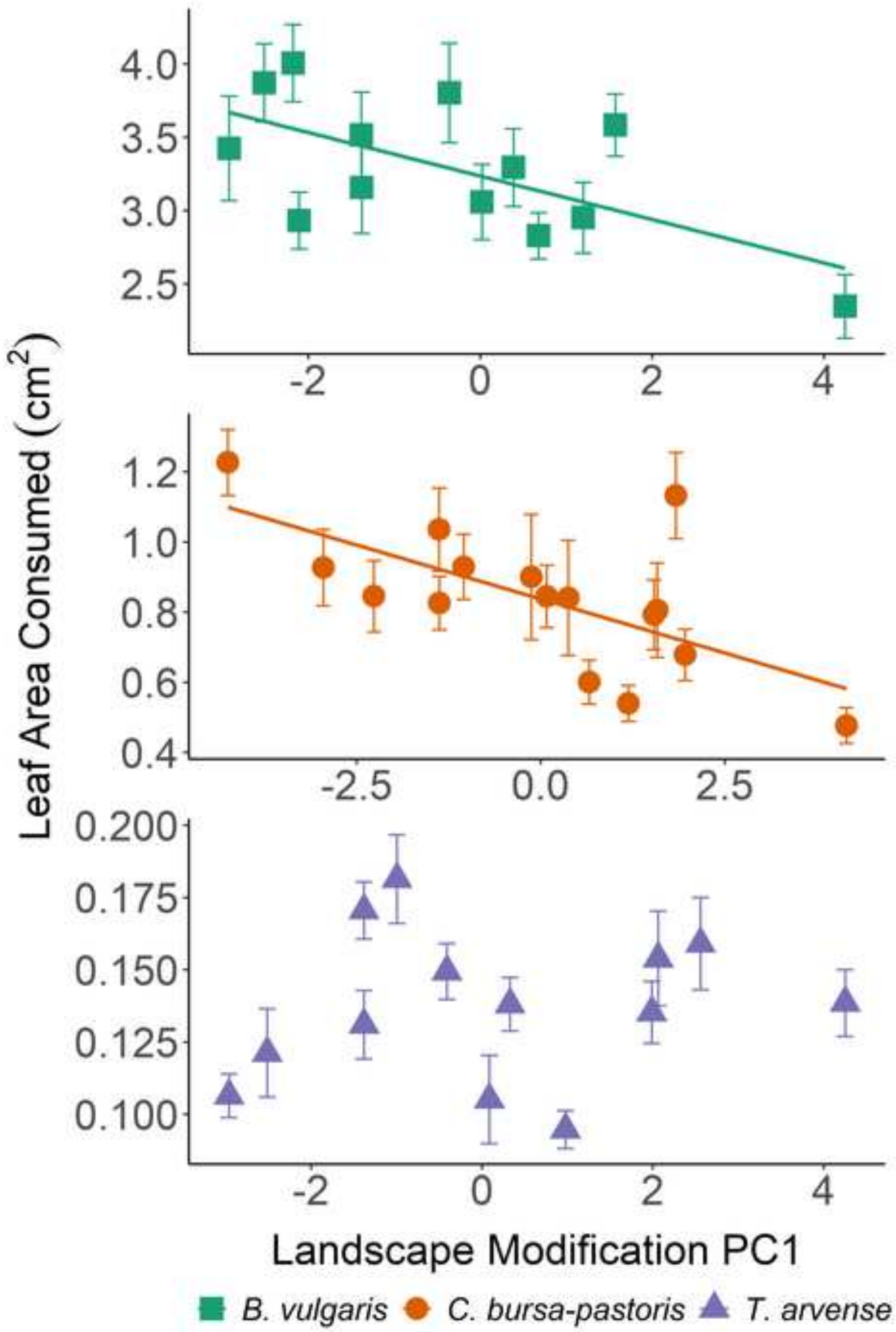

Click here to access/download;Figure;Fig7.tif

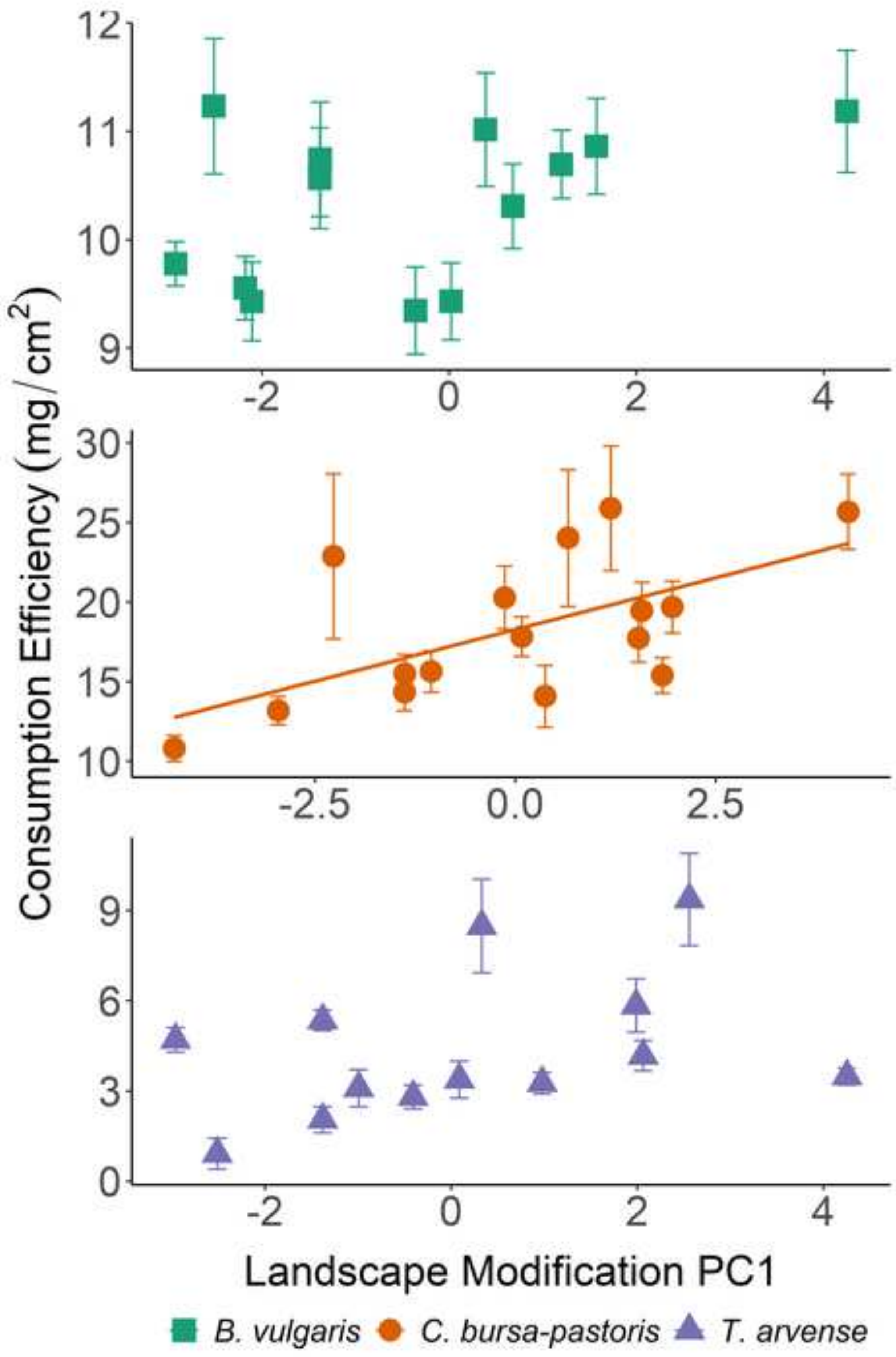

Click here to access/download
Supporting Information
Supporting_Information_PlosOne.docx
