# Supplementary material for: Phenotypic clines in herbivore resistance and reproductive traits in wild plants along an agricultural gradient
Source: PLoS One. 2023 May 31;18(5):e0286050. doi: 10.1371/journal.pone.0286050 (PMC10231797; doi:10.1371/journal.pone.0286050)
Supplement: Attachment — Submitted filename: Response to Reviewers.docx [file pone.0286050.s012.docx]

**RESPONSE TO REVIEWER COMMENTS**

**Schroeder et. al.** **(PONE-D-23-01036)**

**Reviewer 1:**

**The introduction is too general and does not adequately prepare the reader for the rest of the manuscript. I suggest reducing the "General literature" section on the effects of agriculture on plant traits and phenotype and providing more detail on the impact on reproductive traits and herbivory resistance. Consider what the reader needs to be aware of before starting with the description of your experiment, rather than making general statements that do not clearly set the aims.**

Response: We have added a paragraph to the introduction that more explicitly outlines our predictions to better prepare the reader for the specific aims of this manuscript. We believe the general literature section is still necessary to provide context for our specific questions and to highlight the existing work this project is building on.

**Material and Methods:
This section reflects the extensive effort put into the experiment, but I believe it is too long. Consider merging some sections, such as "Study system and landscape analysis," or removing details that are not essential to understanding the process.**

Response: We have removed some non-essential details from the methods, however given the number of traits evaluated across multiple species, many of the details remain necessary for clarity of the study design.

**Results:
I have no general comments to add here. As mentioned in the text, I suggest summarizing Figs. 3-7 into a single 5 x 3 matrix, with each figure as a column. This would greatly improve the interpretability of the work.**

Response: We agree that reformatting the figure will facilitate easier synthesis across plant species and traits. We have combined all trait figures into a single figure with each row representing a trait measured and each column representing a plant species.

**Discussion:
The Discussion section requires clarification. The study produced a lot of information on different plant traits, some influenced by agricultural landscape modification and others by other factors, with varying significance for each species. I recommend to clarify the general conclusions for the reader by creating a scheme or illustration that summarizes all the results and provides a quick overview. Additionally, consider adding a "Final Remarks" or "Conclusions" section at the end of the Discussion to summarize the most important results and those that are most likely to be useful to future researchers.**

Response: By combining all the traits for all species into a single figure we hope to provide the reader with a more cohesive overview of the results. We have also added a “Conclusions” section at the end of the manuscript to summarize the overarching findings.

**Specifics**

**Line 32: I don't think this is the most crucial conclusion that merits to be highlighted from the work**

Response: The text has been modified to read “These findings demonstrate that wild plants are responding to landscape modification, suggesting that the conversion of natural landscapes to agriculture has consequences for wild plant evolution.”

**Line 89: This must be moved to M&M or directly removed. Please, focus on presenting the study species and the aims of the study**

Response: We have removed this methodological information from the introduction.

**Line 93: in terms of herbivore resistance and reproductive traits**

Response: Text modified as suggested.

**Line 97: I recommend to move the information about the study species presented above into this section.**

Response: Because the text cut from the introduction is described in more detail in the methods sections “Seed Collection from Wild Populations,” “Floral Traits,” and “Bioassay,” I simply removed the text from the introduction rather than moving it into the methods.

**Line 99: So they are not native? It is important in terms of evolutionary scale.**

Response: While they are not native, they are able to persist in heavily modified landscapes where most native species are locally extirpated. Additionally, these species have been present in the landscape for over a century and therefore enough time for local adaptation to occur. We have modified the text to read: *While our three study species originate from Eurasia, they have a global distribution and have been present in North America for over a century.*

**Line 105: I would recommend to include literature about the mating systems of the studied species instead of citing personal observation. You would need a robust mating experiment to confirm this.**

Response: We have replaced the reference to personal observation with citations.

**Line 106: Reference needed**

Response: Citations added.

**Line 119: Are these results original from your work? If so, I recommend to add an initial section in Results explaining this. Please note that you provide statistical output before explaining the statistical analysis in M&M**

Response: We have removed this sentence from the methods, added a sentence about the statistical analysis to the methods and moved the statistical output to the beginning of the results section. We have also changed the analysis from a linear regression to a correlation based on comments from Reviewer 2.

**Line 143: I am not involved in this type of fertilizer. Is this a brand? If so, please add ®. Please consider if the provider should be cited as well.**

Response: Jack’s Professional is a brand of fertilizer and so we have added the ® to denote this and added the provider information as well.

**Line 148: Can you add somewhere the starting sample size? How many seedlings were transplanted in total (per species)**

Response: The starting samples sizes per species have been added to the text.

**Line 154: How many?**

Response: The number of *B. vulgaris* plants that were moved to the cooler has been added to the text.

**Line 158: remove “the”**

Response: Corrected as suggested.

**Line 170: For the next experiments I recommend you to use ImageJ software to calculate the precise area of plant structures**

Response: We thank the reviewer for this suggestion. We have modified our methodology in more recent work to measure exact area.

**Line 198: Total sample size per species between brackets**

Response: Total number of plants for each species added to the text.

**Line 209: This citation should be a number according to the journal's bibliography format**

Response: The R Core Team has been added as a citation.

**Line 220: The figure's aesthetic could be improved. Could you try to execute the same PCA but using ggplot aesthetic or similar? At any case, labels should be rewritten with informative names, otherwise it is not clear which variables is indicated in each vector**

Response: We have modified the figure by changing the labels to provide more informative names and ensuring that they do not overlap.

**Line 228: Rewrite: "using the 'lme4' R package (citation)"**

**And include in the References list:**

**Bates D, Mächler M, Bolker B, Walker S (2015). “Fitting Linear Mixed-Effects Models Using lme4.” Journal of Statistical Software, 67(1), 1–48. doi:10.18637/jss.v067.i01.**

Response: Corrected as suggested.

**Line 229: Which kind of models? LM; GLM; GAM...?**

Response: We have modified the text to specify that we ran linear mixed-effects models.

**Line 293: Please rewrite this caption by a more descriptive one which includes all studied species.**

Response: Now that the trait data has been combined into a single figure, this caption is no longer in the manuscript.

**Line 312: You should mention the LMM in M&M (see my comment above)**

Response: Text has been modified to match.

**Line 322: I'd rewrite the caption as: "Herbivory resistance assesment"**

Response: Caption corrected as suggested.

**Line 329: I am not sure about it, once leaves are full expanded, they are not a proxy of leaf age anymore**

Response: Text removed.

**Line 353: effects?**

Response: Corrected as suggested.

**Line 386: Agree. I think that to asses how agricultural landscape is affecting to floral biology you should develop a complete pollination system study. Perhaps you could take the opportunity to include a "further studies" statement about it**

Response: We have added a sentence to address directions for future studies.

**Figure 2: As I mentioned above, vector labels should be replaced by more informative codes**

Response: This figure has been modified to have three simplified vector labels; Proportion Agriculture (500, 1000, and 1500m), Proportion Pasture (500, 1000, and 1500m), and Proportion Natural (500, 1000, and 1500m).

**Figures 3-7: Can Figs. 3, 4, 5, 6 and 7 be merged in a single figure? It would be easier to explain and also visualization would improve**

Response: We agree that reformatting the figure will facilitate easier synthesis across plant species and traits. We have combined all trait figures into a single figure with each row representing a trait measured and each column representing a plant species.

**Reviewer #2**

**This study evaluates intraspecific variation of defense and reproductive traits in 3 plant species according to landscape intensification (increase in proportion of the landscape covered by agriculture).
The topic is very relevant, the experimental methods and analyses are sound and well executed, and the manuscript is clearly written. I feel, however, that the discussion section could be improved. The explanations of the observed patterns and possible mechanisms behind them, fall short after such well-performed study and carefully crafted introduction. I think providing more details about the collecting sites and the landscape profile and history would be very helpful for thinking about potential mechanisms (see below).

Besides that, I have only minor suggestions that may help improve the clarity of manuscript

Specifics:
Introduction
In general, very good and clear introduction.

Line 91: it’s not clear here if reproductive and defense traits were measured in the plants from the field margins or their progeny.**

Response: To clarify this point we have modified the text to read: *To evaluate trait changes in the absence of environmental variation, we collected seeds from plants growing in field margins along a gradient of increasing agricultural area in the surrounding landscape and grew their progeny together in a greenhouse common garden*.

**Methods:
In general, good descriptive section. I do think more details are needed for the collection sites.

Line 112: I think the authors need to provide a detailed description of the collection sites. It would help evaluate the methods as well as provide possible cues for understanding the results. I would like to have information about size and vegetation characteristics of the collecting sites. In addition, a map or a table of the distance between sites, the distribution of the sites along the gradient of landscape intensification, as well as types of agriculture in the buffers (most importantly for this last one, are there Brassica crop fields nearby?).**

Response: We agree that there are a number of interesting questions to ask about specific landscape characteristics, however the goal of this study is to evaluate if agricultural landscape modification on a broad scale influences plant traits. Future studies will be necessary to tackle the specific mechanisms. Further, it is well established in the landscape ecology literature that landscape composition and configuration captures the cumulative effects of these specific landscape characteristics (Martin et al., 2019).

**Line 117: why was a regression used to compare between years and not a correlation matrix? I think the latter would be more appropriate. Related to this, in general, I think there are too many figures. Fig. 1 is not really necessary; presenting the correlation values between years should be enough.**

Response: We re-analyzed the landscape data to obtain Pearson’s correlation coefficients for each year. We believe Fig. 1 is still valuable because it demonstrates the distribution of the sites along the gradient of increasing agricultural landcover.

**Line 127: please explain how plants where “chosen”**

Response: We have modified the sentence to read: *At each site, parent plants were chosen haphazardly in semi-natural patches alongside cultivated crops (field margins, fallow fields, ditches)* **Line 128: this sentence is not clear “The natural variation in landscape composition at the broader scale from 500-1500m around each collection site represented a gradient with an
increasing proportion of agricultural land cover”. Do the authors mean that the sites fell on a landscape gradient? If so, I would like to see a distribution of the sites along this gradient (could be a figure in the supplemental materials or a table with the proportions of agriculture cover for each site).**

Response: We have modified the sentence to read “*Sites were selected such that the* ***existing*** *variation in landscape composition* *at the broader scale from 500-1500m around each collection site represented a gradient with an increasing proportion of agricultural land cover (Fig. 1)”* to highlight that the landscape modification gradient was created by selecting sites based on the composition of the surrounding landscape. The distribution of sites along this landscape gradient is demonstrated in Figure 1.  **Line 182: the sentence that starts at the end of this line is missing a “that”**

Response: Corrected as suggested.

**Line 209: a PCA was run with “combining the most predictive landscape types for
each species across scales”. Please clarify what this means. How did they know in advance which where the ‘most predictive landscape types for each species’?**

Response: In our initial analyses we conducted a dredge to evaluate which landscape type and scale was most predictive across all traits, as explained in the methods. For each species we found a different landscape type and scale was the most predictive (see supplemental figures for these results). To document more generalizable pattern, we conducted a principal component analysis using these three landscape types across all scales.  **Line 220; figure 2: PC2 shows a clear gradient from “natural” to pastures. This gradient seems very relevant for the questions of the study. Why wasn’t it evaluated?**

Response: In the supplemental document we demonstrate that pasture land cover appears to be most important in predicting *T. arvense* trait changes*.* Given we find some of the weakest correlations with PC1 in this species, it is likely that PC2 is a stronger predictor of trait variation for this species. However, we maintain that focusing on PC1 in the main text allows us focus on generalizable patterns associated with agricultural landscape modification specifically. We have also address this by adding the following text to the discussion: *A lack of response by the third species (T. arvense) may be explained by the fact that in the individual analyses, trait variation was best predicted by the proportion pasture in the landscape rather than agriculture.*  **Line 237: Weren’t all plants for this species vernalized?**

Response: In the Germination and Propagation section of the methods we report that 72 *T. arvense* plants bloomed without vernalization and the remaining 210 were vernalized.  **Line 242: sentence starting in this line is not clear.**

Response: We have modified the sentence to read: *Given there was low mortality that did not correlate with the landscape gradient, we excluded any caterpillars that did not survive to the end of the experiment in all future models.*  **Line 246: please briefly explain what ‘bolting status’ is and why was it important to include it as random effect.**

Response: We have modified the text to read: *we included bolting status (a binary variable describing whether or not a plant had developed reproductive structures as the time of the experiment) as a random effect for T. arvense and C. bursa-pastoris to account for variation in plant ontogeny.*

**Results
In general, the results are clearly presented but I do think the figures could be used more efficiently (see below)

256: please replace “higher agricultural landscapes” with a more descriptive term and use it consistently across the manuscript. The authors also use ‘landscape modification’, ‘increasing agricultural habitat modification’ and other terms to refer to the ‘increasing amount of agriculture cover in the landscape’. Use one consistent term across the manuscript.**

Response: We have modified the text to use “agricultural landscape modification” consistently throughout the manuscript. **261: These are regression coefficients (GLMMs) not correlation coefficients**

Response: Correlations changed to regression coefficients.  **Figures 3-7. Just a suggestion but I think these figures could be combined into one summary figure that would make it easier to see the found patterns. As they are now, it’s hard to contrast between the different reproductive and defense traits. The summary figure could just give information of the direction of the effects (+/-/0) between increasing ag. cover and each of the different traits (somehow separated by ‘defense’ ‘reproductive’). The detailed regression figures could be provided in the supplemental material.**

Response: We have combined all trait figures into a single figure with each row representing a trait measured and each column representing a plant species. **Discussion:
In general, I think the discussion was the weakest section of the manuscript. I would suggest a more careful interpretation of the results. More information on the sites, on the natural history of the plants (and the plants surrounding them), on the history of the sites, may provide cues for the potential mechanism behind the results.**

Response: We have added an additional “Conclusions” section at the end of the manuscript based on comments form Reviewer 1. We hope this aids in clarifying the overall patterns observed in this study. As for the specific information about the collection sites, the goal of this study was to examine whether the composition of the surrounding landscape broadly predicts plant trait changes. Future studies will now need to test the specific mechanisms. We highlight examination of the changes in insect interactions as one key area for future studies.  **379: given that the authors are talking about selection pressures here, it would be worth it to, at the very least, mention how long these landscapes have been agriculture dominated (10 years may not be enough).**

Response: Given work in another Brassicaceae species, *Brassica rapa*, demonstrating trait adaptation in as little as 8 generations, ten years is likely enough time for to observe trait shifts in rapid cycling species (Ramos & Schiestl, 2019). Additionally, the region of New York state where this study was conducted has been agricultural for the past 100 years. Our quantitative analysis on the last ten years shows that there have not been recent major land use changes.

**387: The explanation and interpretation for the results in this paragraph are not clear. It is not clear what the authors are concluding from these results: “caterpillars consumed less leaf area and developed more efficiently on plants from agriculturally dominant landscapes.” But given this sentence “Studies examining plant defenses in urban landscapes have found similar results to those presented here, demonstrating a decrease in plant defenses within modified urban landscapes (21,52)” tells me that the authors are saying that their results suggest decreased plant defenses in ag. dominated landscapes. I’m not sure why. Caterpillars consuming less leaf area and developing more efficiently means (or suggests) that the plants in ag. dominated areas are better defended, not less. This is not discussed in this paragraph.**

Response: We agree that this interpretation is more accurate and have modified the text to reflect this.
